# Using expert knowledge to incorporate uncertainty in cause‐of‐death assignments for modeling of cause‐specific mortality

**DOI:** 10.1002/ece3.3701

**Published:** 2017-11-30

**Authors:** Daniel P. Walsh, Andrew S. Norton, Daniel J. Storm, Timothy R. Van Deelen, Dennis M. Heisey

**Affiliations:** ^1^ National Wildlife Health Center U.S. Geological Survey Madison WI USA; ^2^ Department of Forest and Wildlife Ecology University of Wisconsin‐Madison Madison WI USA; ^3^ Wisconsin Department of Natural Resources Bureau of Science Services Rhinelander WI USA; ^4^ Present address: 35365 800th Avenue Madelia MN 56062 USA

**Keywords:** cause‐specific mortality, expert elicitation, hazard, *Odocoileus virginianus*, regularization, survival analysis, time‐to‐event, uncertainty

## Abstract

Implicit and explicit use of expert knowledge to inform ecological analyses is becoming increasingly common because it often represents the sole source of information in many circumstances. Thus, there is a need to develop statistical methods that explicitly incorporate expert knowledge, and can successfully leverage this information while properly accounting for associated uncertainty during analysis. Studies of cause‐specific mortality provide an example of implicit use of expert knowledge when causes‐of‐death are uncertain and assigned based on the observer's knowledge of the most likely cause. To explicitly incorporate this use of expert knowledge and the associated uncertainty, we developed a statistical model for estimating cause‐specific mortality using a data augmentation approach within a Bayesian hierarchical framework. Specifically, for each mortality event, we elicited the observer's belief of cause‐of‐death by having them specify the probability that the death was due to each potential cause. These probabilities were then used as prior predictive values within our framework. This hierarchical framework permitted a simple and rigorous estimation method that was easily modified to include covariate effects and regularizing terms. Although applied to survival analysis, this method can be extended to any event‐time analysis with multiple event types, for which there is uncertainty regarding the true outcome. We conducted simulations to determine how our framework compared to traditional approaches that use expert knowledge implicitly and assume that cause‐of‐death is specified accurately. Simulation results supported the inclusion of observer uncertainty in cause‐of‐death assignment in modeling of cause‐specific mortality to improve model performance and inference. Finally, we applied the statistical model we developed and a traditional method to cause‐specific survival data for white‐tailed deer, and compared results. We demonstrate that model selection results changed between the two approaches, and incorporating observer knowledge in cause‐of‐death increased the variability associated with parameter estimates when compared to the traditional approach. These differences between the two approaches can impact reported results, and therefore, it is critical to explicitly incorporate expert knowledge in statistical methods to ensure rigorous inference.

## 1 INTRODUCTION

Expert knowledge is increasingly being recognized as an important source of information in ecological modeling for management, research, and policy development (Drescher et al., 2013; Kuhnert, Martin, & Griffiths, 2010) and is particularly valuable when other empirical sources of information are lacking (e.g., emergence of new infectious disease or rare species’ habitat preferences). Development of methods incorporating expert knowledge into statistical models has blossomed (Albert et al., 2012; Low Choy, O'Leary, & Mengersen, 2009), especially within the Bayesian paradigm that inherently views probability as a measure of uncertainty (Gelman et al., 2014; Lindley, 2000). This interpretation facilitates a rigorous approach to integrating expert knowledge via prior distributions (Albert et al., 2012; Low Choy et al., 2009).

Use of expert knowledge has been criticized as subjective and unrepeatable (Cox, 2000; Drescher et al., 2013; Johnson & Gillingham, 2004; Kuhnert et al., 2010). However, expert knowledge often is used implicitly in ecological studies, although typically not recognized as such. Instances of implicit use generally arise when an outcome of interest cannot be assessed with certainty, and response variables are assigned based on a researcher's judgment. This assigned response value is then treated as a fixed, known value with analytical procedures proceeding in standard fashion, while ignoring the implicit use of expert knowledge and potential inherent uncertainty associated with the observer's assignment. However, to properly account for the uncertainty during analyses and model selection procedures, and to ensure proper inference is made regarding the hypotheses of interest, the researcher's uncertainty associated with each of their assigned responses should be quantified and incorporated into estimation procedures.

To demonstrate the importance of explicitly rather than implicitly incorporating expert knowledge into statistical developments, we focus on estimation of cause‐specific mortality. Ecological studies investigating cause‐specific mortality typically rely on capturing and marking individuals with tracking transmitters (Heisey & Patterson, 2006; Pollock, Winterstein, Bunck, & Curtis, 1989; Walsh, Dreitz, & Heisey, 2015) that allow researchers to monitor animals and identify time of death. Researchers investigate these mortality events and assign a cause‐of‐death based on their expert knowledge. These types of studies represent implicit use of expert knowledge because cause‐of‐death assignments are associated with varying degrees of certainty. For example, cause‐of‐death of a hunter‐killed animal may be considered known when reported to the researcher or observed directly, whereas there may be large uncertainty associated with cause‐of‐death in other instances (e.g., distinguishing between predation and starvation followed by scavenging). In this latter case, simply choosing a “best guess” of cause‐of‐death based on the researcher's knowledge can lead to misclassification, potential bias in the cause‐specific hazards, an underestimation of the variance of parameter estimates, and incorrect model selection results (Liberg et al., 2012; Moreno‐Betancur & Latouche, 2013; Van Rompaye, Jaffar, & Goetghebeur, 2012). To provide more accurate estimate of cause‐specific hazards, and explicitly model expert knowledge regarding uncertainty in cause‐of‐death assignments, we extend current statistical modeling approaches (Cross et al., 2015) to incorporate uncertainty in cause‐of‐death assignments using a data augmentation (Tanner & Wong, 1987) approach based upon prior predictive values (PP) elicited from the researcher when they assigned each cause‐of‐death.

Our objectives were to (1) describe an approach for including expert knowledge regarding uncertainty of event type into a hierarchical model of cause‐specific and baseline hazards, (2) simulate data to demonstrate performance of model with uncertainty including effects on accuracy and precision, (3) predict annual cause‐specific mortality outside the hunting season for white‐tailed deer using our hierarchical modeling framework, and 4) compare parameter estimates using our approach with those from the standard approach.

## 2 METHODS

Our approach to estimate cause‐specific mortality expands on the two‐component model of Cross et al. (2015). For the first component, we modeled the overall event hazard using the conditional survival function (Kalbfleisch & Prentice, 1980) where we defined the overall event hazard as any death irrespective of the source of mortality, excluding known censoring events (e.g., survived to the end of study, dropped collar). The likelihood contribution of the *i*th subject was as follows:

$$\text{Pr}\left(r\leq T_{i}<s|T_{i}\geq e\right)=\text{exp}\left(-\int_{e_{i}}^{r_{i}}h\left(t\right)dt\right)\left[1-\exp\left(-\int_{r_{i}}^{s_{i}}h\left(t\right)dt\right)\right],$$

where *T*_*i*_ = time of death; *e*_*i*_ = time of entry; *r*_*i*_ = time that the subject was last known to be alive; *s*_*i*_ = time subject was first encountered dead; and *h* (*t*) = instantaneous hazard. This formulation allows left‐truncated and interval‐censored data. Under the assumption of independence of individual fates, likelihood contributions were multiplied across subjects.

For estimation, we assumed *r* and *s* were integers, and approximated the cumulative hazard function using a piece‐wise constant hazard model: $\int_{r}^{s}h(t)dt=\sum_{r}^{s}\Lambda_{u},$ where the unit cumulative hazard, $\Lambda_{u}=\int_{r}^{r+1}h(t)dt.$ We focused our modeling efforts on the natural log of the unit cumulative hazard, and incorporated covariates as follows: ln (*Λ*_*i*,*u*_) = γ_*u*_ + β_*j*,*u*_ *x*_*i*,*j*,*u*_ ln (*Λ*_*i*,*u*_) = γ_*u*_ + β_*j*,*u*_ *x*_*i*,*j*,*u*_, where γ_*u*_ represents the baseline log cumulative hazard for the *u*th interval; *x*_*i,j,u*_ **= the *j*th covariate for the *i*th subject during the *u*th interval; and β_*j*,*u*_ = is the effect of the *j*th covariate during the *u*th interval and is the log hazard ratio. This formulation closely follows the stepwise generalized linear model approach using the complementary log–log link or the discrete data proportional hazards model (Heisey, Shaffer, & White, 2007; Heisey et al., 2010; Prentice & Gloeckler, 1978).

To account for the competing risks nature of various potential sources of mortality, we partitioned the overall event hazard by each source of mortality in the second component of our model. Conditional on death, we used the categorical distribution to estimate the probabilities the death was due to the various potential sources of mortality, hereafter called cause‐specific probabilities (Cross et al., 2015; Figure S1). The probability an individual's death was associated with a specific source of mortality (π_*k*_) was modeled as: cause_*i*,*u*,*k*_ ~ Categorical[π_*u*,*k*_], where cause_*i*,*u*,*k*_ =  indicator (1 or 0) if cause‐of‐death for the *i*th subject during the *u*th interval was assigned by the observer to the *k*th source of mortality. Additionally, as described below in our applied example, covariate information can be incorporated via the multinomial logit model:

$$\pi_{i,u,k}=\left[\frac{\exp(x_{i,u}^{\prime }\beta )}{\sum_{k=1}^{K}\exp(x_{i,u}^{\prime }\beta )}\right],$$

where $x_{i,u}^{\prime }$ is a row vector of covariates and **β** is the vector of parameters.

Thus, combining our model components yields the following joint probability that a subject was alive through interval *U* − 1, died during interval *U*, and the cause‐of‐death was assigned to the *k*th source of mortality:

$$\mathrm{Pr}\left(t<T_{i}<t+\Delta ,K=k|T_{i}>t\right)=\psi_{i,k,u}=\exp\left(-\sum_{u=1}^{U-1}\Lambda_{i,u}\right)\times \left[1-\exp\left(\Lambda_{i,U}\right)\right]\times \pi_{i,u,k}.$$

### 2.1 Accounting for uncertainty in cause‐of‐death assignment

In the final step in model development, we incorporated observers’ expert knowledge regarding likely cause‐of‐death for each dead individual. Within the Bayesian paradigm that views probability as a measure of uncertainty, we extended our two‐component model by eliciting the observer's knowledge, via PP, that each potential source of mortality was the true cause‐of‐death. Our approach follows classical misclassification theory (Hoenig, Hanumara, & Heisey, 2002) that involves two sets of “atomic” parameters from which quantities of interest are constructed. These sets are referred to as (1) the misclassification parameters that describe the probability of an observer assigning a cause‐of‐death conditional on the true cause‐of‐death and (2) prevalence parameters that describe the true probability of dying due to each potential source of mortality. Misclassification appears to have received its first applications in epidemiological disease testing where misclassification parameters generalize the binary epidemiological concepts of test sensitivity and specificity.

In our model, we assumed a mortality, *m*, was randomly selected and the source of mortality is identified with an integer value, say 1,…,*K*. Each mortality had two associated random variables: (1) the true fate, *A*_*m*_ = *i* that was latent/unobserved and (2) *E*_*m*_ = *j* was the error‐prone classification by the observer. We defined each prevalence parameter as $\mathrm{Pr}\left(A_{m}=i\right)=\pi_{i}$ that was the cause‐specific fate proportion. The misclassification parameters were defined as $\mathrm{Pr}\left(E_{m}=j|A_{m}=i\right)=\phi_{\mathrm{ij}}$, and for a fixed *i*, these sum to 1 across *j*. Each of these parameters represents the probability, given the latent fate is *i*, and the observer assigned the *m*th mortality to source *j*. They are nuisance parameters that must be accommodated to prevent inaccurate estimation of parameters of biological interest (i.e., prevalence). The remainder of our model is constructed from these prevalence and misclassification parameters.

Our framework is based on the assumption that the observer uses evidence at the death site to make their best guess regarding source of mortality, *E*_*m*_. Thus, although *E*_*m*_ denotes an error‐prone assignment, it can also be thought of as representing the summary of death site evidence. Thus, we argue the PPs are the summary of the observers’ best guesses of the probability the true fate *A*_*m*_ was of type *i*, given the observer concluded *E*_*m*_ = *j* based on the death site evidence. More formally, the PPs are guesses of $\eta_{\mathrm{ij}}=\mathrm{Pr}\left(A_{m}=i|E_{m}=j\right)$. In epidemiology, such quantities are referred to as “predictive values” because they represent the value of observed quantities (test results) for predicting the true biological state of interest. The joint distribution of the combinations of the bivariate categorical variables, $\mathrm{Pr}\left(A_{m}=i,E_{m}=j\right)$, is given as:

$$\Gamma =\left[\begin{array}{cccc} {\pi_{1}\phi }_{11} & {\pi_{2}\phi }_{12} & {\pi_{3}\phi }_{13} & {\pi_{4}\phi }_{14}\\ {\pi_{1}\phi }_{21} & {\pi_{2}\phi }_{22} & {\pi_{3}\phi }_{23} & {\pi_{4}\phi }_{24}\\ {\pi_{1}\phi }_{31} & {\pi_{2}\phi }_{32} & {\pi_{3}\phi }_{33} & {\pi_{4}\phi }_{34}\\ {\pi_{1}\phi }_{41} & {\pi_{2}\phi }_{42} & {\pi_{3}\phi }_{43} & {\pi_{4}\phi }_{44} \end{array}\right].$$

Prior predictive values can be constructed from *Γ* by simply normalizing the columns of the *j*th row to sum to 1:

$$\eta_{\mathrm{ij}}=\mathrm{Pr}\left(A_{m}=i|E_{m}=j\right)=\frac{\mathrm{Pr}\left(A_{m}=i,E_{m}=j\right)}{\mathrm{Pr}\left(E_{m}=j\right)}=\frac{{\pi_{i}\phi }_{\mathrm{ij}}}{\sum_{k=1}^{K}{\pi_{k}\phi }_{\mathrm{kj}}}.$$

Of course, it would be preferable for observers to have reported the true fate *A*_*m*_, but this is often infeasible. Therefore, in lieu of observing *A*_*m*_'s directly, a correction was necessary to the *E*_*m*_'s to estimate prevalence. Assuming mortality *m* was drawn randomly, the *E*_*m*_'s are representative of the population distribution of *E*_*m*_'s, and if the expected value of $\mathrm{Pr}\left(A_{m}=i|E_{m}=j\right)$ is taken with respect to the *E*_*m*_, we obtain the expected value of *A*_*m*_, or the prevalence. This is the justification of our data augmentation‐like approach, described below, where we drew random samples from the PP distribution that we assume represents an approximation to $\mathrm{Pr}\left(A_{m}=i|E_{m}=j\right)$. This provided a more accurate approximation of the “true” data *A*_*m*_ than using the error‐prone assignments, *E*_*m*_. Our simulations test this theory using a rather conservative approach to generating reasonable PPs.

To provide an example of the implementation of the elicitation process, assume an observer believed *E*_*m*_ = “source 1.” We then elicited the likelihood of each mortality source being the cause‐of‐death given *E*_*m*_ = “source 1,” for which the observer assigned $\eta_{1,1}=0.75$ and $\eta_{2,1}=0.25$ for the two potential mortality sources. This provided the vector, PP = [0.75, 0.25] that inherently quantified their uncertainty of the true cause‐of‐death, *A*_*m*_. If the observer was completely certain, “source 1” was the cause‐of‐death they would specify $\eta_{1,1}=1$, and all remaining $\eta_{i,j}=0$.

Once we had elicited observer‐specified PP for each mortality, we used data augmentation methods (Tanner & Wong, 1987) within an MCMC algorithm to estimate overall event hazard and cause‐specific probabilities, while incorporating observer‐specified PP. We treated true cause‐of‐death for each individual as an unobserved, latent variable, and during each MCMC iteration, this cause‐of‐death was drawn from a categorical distribution whose parameters are specified by the PP for that individual.

In summary, our statistical approach uses a two‐component model to estimate overall event hazard rate and cause‐specific probabilities, and incorporates expert knowledge to quantify the uncertainty of true cause‐of‐death. Implementing this model within a Bayesian hierarchical framework permits a simple and rigorous estimation method that can be modified easily to include covariate effects and regularizing terms. Lastly, although formulated in terms of survival analysis, this approach is applicable to any event‐time analyses where multiple types of events are possible, there is uncertainty in the event type, and expert knowledge can be elicited regarding the type of event.

### 2.2 Simulations

We evaluated the performance of our statistical approach relative to current techniques via simulation. We simulated mortality datasets for 50 individuals, and we generated failure times for each individual using an exponential distribution with a rate of 0.1. We set our study length to 36 months, and individuals whose failure time exceeded the length of the study were considered right‐censored. We determined the observer‐assigned cause‐of‐death for each individual mortality using unequal probability sampling where the sampling weight for the *j*th potential sources of mortality, Pr(*E*_*m*_ = *j*), was the sum of *j*th row of Γ. Next using unequal probability sampling with sampling weights = $\eta_{\mathrm{ij}}$, as described above, we determined true cause‐of‐death conditional on the observer‐assigned cause‐of‐death. Lastly, we created the vector of PP for each mortality. We generated these values from a Dirichlet distribution with parameters equal to the *j*th row of Γ, corresponding to the observer‐assigned cause‐of‐death determined previously. This distribution was constrained such that the *j*th element of the PP vector was the maximum element. This constraint is needed to ensure the observer‐assigned cause‐of‐death will have the largest PP. We implemented the constraint using a naïve rejection sampler (Robert, 1995).

To assess the influence of varying the prevalence (π_*i*_) and misclassification parameters ($\phi_{\mathrm{ij}}$), we evaluated four different patterns of mortality by setting the vector of prevalence parameters to one of the following: (1) [0.4, 0.3, 0.2, 0.1]; (2) [0.1, 0.2, 0.3, 0.4]; (3) [0.45, 0.45, 0.05, 0.05]; or (4) [0.7, 0.1, 0.1, 0.1]. We also created nine confusion/misclassification matrices where the diagonal elements (i.e., probability of correctly assigning cause‐of‐death) were equal and ranged from 0.25 to 1.00, and off‐diagonal elements were all equal, indicating if cause‐of‐death was incorrectly assigned, each remaining source of mortality was equally likely to be assigned as the cause‐of‐death (see Supporting Information). Additionally, we created a classification matrix to represent the common field situation where the first two mortality sources are more likely to be confused with each other, while the remaining sources of mortality tend to be correctly classified. The classification matrix (C10) we used for this scenario was as follows:

$$\left[\begin{array}{cccc} 0.5 & 0.4 & 0.05 & 0.05\\ 0.4 & 0.5 & 0.05 & 0.05\\ 0.0\overset{¯}{6} & 0.0\overset{¯}{6} & 0.8 & 0.0\overset{¯}{6}\\ 0.0\overset{¯}{6} & 0.0\overset{¯}{6} & 0.0\overset{¯}{6} & 0.8 \end{array}\right].$$

Lastly, we created a classification matrix where the first source of mortality may be confused with a second, but the second is correctly classified at a high rate, and remaining sources tend to be correctly classified. The associated classification matrix (C11) was as follows:

$$\left[\begin{array}{cccc} 0.5 & 0.4 & 0.05 & 0.05\\ 0.2 & 0.7 & 0.05 & 0.05\\ 0.0\overset{¯}{6} & 0.0\overset{¯}{6} & 0.8 & 0.0\overset{¯}{6}\\ 0.0\overset{¯}{6} & 0.0\overset{¯}{6} & 0.0\overset{¯}{6} & 0.8 \end{array}\right].$$

We generated 500 datasets under each combination of the prevalence and misclassification parameters.

Once we generated data, we estimated the hazard and probability of dying from the four sources of mortality using current Bayesian approaches that assume no uncertainty in the assignment of the cause‐of‐death (Cross et al., 2015), our approach that includes expert knowledge via observer prior distributions as described above, and compared them to a model in which observers correctly assigned cause‐of‐death. For each model's parameters, we created three MCMC chains, and ran them for 15,000 iterations with a 1,500 iteration burn‐in period that in preliminary analyses did not exhibit evidence of nonconvergence. We used a normal (0, 10^6^) prior for the natural log of the hazard rate, and we specified the parameters for the Dirichlet prior distribution = [1, 1, 1, 1] for the vector of multinomial probabilities associated with dying from each source of morality under the current approach. We repeated the above data and estimation procedures for each dataset, and compared these modeling approaches by evaluating percentage relative bias, standard deviation, and credible interval coverage for each parameter across datasets. All simulations were conducted in JAGS (Plummer, 2003) via the statistical program R (R Development Core Team, 2016) in conjunction with the R2JAGS library (Su & Yajima, 2015). The algorithms we used to conduct the simulations and a detailed description of them are included in the Supporting Information.

### 2.3 Applied example: cause‐specific hazard models

We used our statistical approach to estimate cause‐specific mortality rates of white‐tailed deer (*Odocoileus virginianus*) outside the hunting season from 2011 to 2014 across a 5,905‐km^2^ study area in the northern forest region of Wisconsin, USA. We fitted captured deer with VHF radiocollars (Advanced Telemetry Systems Inc., Minnesota, USA) and monitored all radiocollared deer survival 1–3 times per week. We immediately censored deer, determined to be lost to follow‐up independent of fate, after their last known alive telemetry signal was confirmed. All animal handling was approved by University of Wisconsin Animal Care and Use Committee (Research Animal Resources Center, protocol number A01446).

Mortalities were classified into three possible sources of mortality: predation, human‐associated causes, and all other causes. To determine the PP vector for possible mortality sources when a collar signaled mortality, trained field personnel conducted a site investigation and, if possible, a carcass necropsy. Additionally, they documented time of year, recent temperature, snow cover, and local predator abundance to help inform potential fate determination and assignment of PP values. All information was recorded on datasheets. We then interviewed field personnel and reviewed datasheets to assign PP when fate was uncertain. Specifically to standardize assignment of PP, we a *priori* developed a set of guidelines for assigning points toward each of the three causes based on confidence in each cause. For example, when assigning points for wolf predation, we initially assigned 90 points if we were confident in the cause, 70 points if the cause was likely, 30 points if there was some chance, and 10 points if there was a slight chance the cause was indeed the fate. These four levels of points could also be increased or decreased from their initial values if these starting point values were not sufficient to reflect our confidence in a cause based on the evidence at the site. We then summed the points across all causes that were grouped into our three possible sources of mortality. For example, for predation, we summed all the points associated with each type of predation (i.e., wolf, coyote, bobcat, other). Once points had been assigned for all three possible sources of mortality, we standardized the points by dividing the cause‐specific score by the total among all three possible sources of mortality, providing the PP vector (see Appendix S1 for detailed protocol). In the event, we were not confident in the assigned PP, we re‐visited the findings with a group of professionals (e.g., field biologists, predator and deer research scientists, veterinarians), and adjusted the PP associated with that individual, as described above.

For estimation, we pooled all data from radiocollared deer ≥7 months old collected from 1 day immediately following to 1 day immediately preceding the hunting season, and treated information for each individual as independent across years. Data used for the overall hazard analysis consisted of encounter histories with a single record for every four weeks (monthly; nine total intervals) the deer was available. If a deer was captured during an interval, it was considered available if the capture date occurred during the first half of the interval.

To estimate the hazard of dying outside the hunting season and associated cause‐specific probabilities, we examined several models with additive age and study year effects (AGE_add_, YEAR_add_). Our full, additive overall hazard model was as follows: γ_*u*,year,age_ = γ_0,*u*_ + β_year_ + β_age_, where γ_*u*,year,age_ = interval‐specific log hazard for each year and age group and γ_0,*u*_ = intercept for each interval. To share information and borrow strength across intervals, we regularized γ_0,*u*_ using the following hierarchical structure: $\gamma_{0,u}∼N\left(\gamma_{0},\sigma^{2}\right)$, where $\gamma_{{}_{0}}∼N\left(0,100^{2}\right)$ and $\sigma ∼\text{Uniform}\left(0,10\right)$. We also investigated age interaction models (AGE_int_) and year interaction models (YEAR_int_). Within the age interaction model, we replaced γ_0,*u*_ with a separate interval intercept for each age group, γ_0,*u*,age_,  and it was regularized in the same manner described for γ_0,*u*_. We used age interaction models to investigate the hypothesis: Survival of juveniles (~7–15 months old) may be more sensitive to annual variation than adults (>19 months old). Similarly, we constructed interaction models for each year of study. Lastly, we examined a model with γ_0,*u*_ being the sole parameter, and it was regularized as described above and we refer to hereafter as the “BASELINE” model. Example BUGS code is provided in the Supporting Information, and model descriptions are provided in Figures S2 and S3.

To keep our suite of biologically relevant models small, we used a stepwise model selection procedure to select among competing models for the overall hazard of dying (Burnham & Anderson, 2002). First, we selected among different additive and interaction year or age models, separately. We then combined parameters from best‐fit age and year models (if they had better fit than the BASELINE model) and compared against age‐ or year‐only models. We evaluated model fit using DIC values and selected the most parsimonious model, based on lowest DIC value, from which to make an inference.

We constructed the same suite of models for each cause‐specific probability. This ensured that we could assess covariate effects on each potential source of mortality separately. For example, using the multinomial logit model, we evaluated the variation among study areas and years: $\phi_{u,k}=\phi_{0,u,k}+x_{i,u,k}^{\prime }\beta_{u,k}$ We regularized ϕ_*u*,*k*_ for each cause across intervals:

$$\phi_{0,u,k}∼N\left(\phi_{0,k},\sigma_{k}^{2}\right).$$

We specified priors for the hyperparameters as:

$$\phi_{0,k}=\ln\left(\frac{\rho_{k}}{\rho_{j}}\right),$$

$$\rho_{k}=\frac{g_{k}}{\sum_{i=1}^{K}g_{i}},$$

$$g_{k}∼\text{Gamma}\;\;\left(\alpha_{k},\alpha_{k}\right),$$

$$\sigma_{k}∼\text{Uniform}\left(0,10\right)\;,$$

where ρ_*j*_ represents conditional probability of death due to predation. We used the gamma distribution to specify a Dirichlet prior for $\vec{\rho }$ (Lunn, Jackson, Best, Thomas, & Spiegelhalter, 2013). We used a diffuse prior with values of 1 for α_*k*_. We used the same model selection procedure for models of cause‐specific probabilities as described above.

During each MCMC iteration, we included expert knowledge using the elicited probabilities of the likely cause‐of‐death (PP) by augmenting the causes‐of‐death that were presumed known with random realizations from the $Categorical\left(PP_{u,k}\right)$ distributions for those mortalities where cause‐of‐death was uncertain. These augmented causes were then used to model cause‐specific probabilities (i.e., $causeaug_{i,u,k}∼Categorical\left(\pi_{u,k}\right)).$)

To emulate current models and compare approaches, we assumed cause of mortality was known with certainty for all mortalities by treating the cause‐of‐death with the highest probability as the “known” cause. We compared results from models including uncertainty to those that assumed the cause‐of‐death was known.

Similar to our simulation analysis, we created three MCMC chains and ran each chain for 15,000 iterations. However, we used a 10,000 iteration burn‐in period that based on standard diagnostics provided no evidence of nonconvergence. We conducted all MCMC algorithm analyses for our applied example using WinBUGS (Spiegelhalter, Thomas, & Best, 2002) in conjunction with R2WinBUGS library (Sturtz, Ligges, & Gelman, 2005).

## 3 RESULTS

### 3.1 Simulations

Our model outperformed current approaches with lower bias and higher credible interval coverage of parameters, regardless of the true probabilities of cause‐of‐death/prevalence rates or probability of correctly assigning the cause‐of‐death/misclassification rates (Figures S4–S13). The one exception was for classification matrix C11 where confidence interval coverage for the parameter for the second cause‐of‐death was slightly less. As expected, our model generally increased the standard deviation of all parameters across all simulations compared to current approaches. Additionally, as misclassification decreased, our model more rapidly approached the performance of the model for which there was no misclassification error.

### 3.2 Applied example

We used information from 433 unique, radiocollared deer ≥7 months old to evaluate mortality outside the hunting season between 2011 and 2014. Because deer that survive can be used for subsequent years, we evaluated more encounter histories than the number of unique, radiocollared deer (Van Deelen, Campa, & Haufler, 1997). We included encounter history sample sizes and fate information in Table 1. There were 57 of 125 mortality events that included uncertainty in fate determination. The average user‐specified PP for these events was 0.106 for human causes, 0.563 for predation and 0.331 for other causes.

**Table 1:** Sample sizes for radiocollared white‐tailed deer available and cause‐specific events (based on the cause with the highest observer‐specified probability) by age and year for survival outside the hunting season. Parenthetical information is the sum of event probabilities based on expert knowledge. Three different sources of mortality were investigated, namely human‐caused mortality (human), predation events (predation), and all other causes (other)

Age (months)	Year	Available	Human	Predation	Other
7–15	2011	44	2 (2.00)	9 (8.150)	3 (3.850)
7–15	2012	31	1 (1.00)	0 (0.000)	0 (0.000)
7–15	2013	68	3 (3.68)	14 (12.320)	5 (6.000)
7–15	2014	103	2 (1.95)	30 (27.227)	8 (10.823)
>19	2011	61	4 (3.90)	5 (4.750)	2 (2.350)
>19	2012	87	1 (1.20)	9 (7.900)	1 (1.900)
>19	2013	93	1 (1.00)	7 (6.700)	5 (5.300)
>19	2014	87	3 (2.30)	8 (8.050)	2 (2.650)
All	2011–2014	574	17 (17.030)	82 (75.097)	26 (32.873)

**Figure 1:**
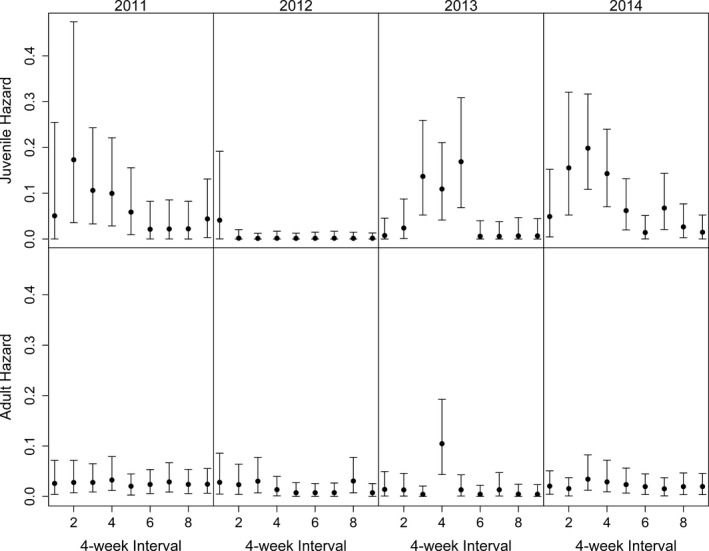
Regularized estimates of the hazard of dying with 95% credible intervals for each 4‐week interval (*N* = 9) for hazards outside the hunting season for juveniles (~7–15 months old) and adult (>19 months old) white‐tailed deer from 2011 to 2014 in Wisconsin, USA, using a model with independent year and age effects

Based on DIC values, the overall hazard model, component 1 of our model, best supported by the data included an interaction term for year and age group (Table 2). Parameter estimates for the log of the overall hazard of dying suggested lower survival with greater variation among monthly intervals for juveniles (7–15 months old) compared to adults in 2011, 2013, and 2014. In 2012, adults had a higher average monthly hazard than juveniles (Table 3, Figure 1).

**Table 2:** Log hazard models of Wisconsin white‐tailed deer from 2011 to 2014. Models allowed for a time‐varying baseline with nine monthly intervals based on an interval regularizing parameter. The BASELINE model only estimated interval‐specific parameters assuming no difference among adult and juvenile deer (AGE) and YEAR. Subscripts add estimated additive differences among age classes or years and int estimated independent age or year effect

Model^a^	DIC	pD	Δ DIC
AGE_int_ + YEAR_int_	977.69	40.01	0.00
AGE_int_	998.00	14.63	20.32
AGE_add_	1001.58	9.19	23.90
YEAR_int_	1016.42	26.52	38.74
YEAR_add_	1017.72	11.22	40.04
BASELINE	1030.64	8.16	52.96

a Additional model description included in Figure S3.

**Table 3:** Parameter estimates for log hazard models including an independent year and age effects for Wisconsin white‐tailed deer from 2011 to 2014. Model included a regularizing parameter for different log hazards among nine monthly intervals each year, different for juveniles (~7–15 months old; $N(\gamma_{0,year,juvenile},\sigma_{year,juvenile}^{2})$) and adults (>19 months old; $N(\gamma_{0,year,adult},\sigma_{year,adult}^{2})$). All parameters are on the log hazard scale

Parameter^a^	Mean	*SE*	2.50%	97.50%
γ_0,2011,juvenile_	−3.559	1.124	−6.460	−2.173
σ_2011,juvenile_	1.816	1.406	0.228	5.735
γ_0,2012,juvenile_	−11.757	5.120	−23.890	−4.970
σ_2012,juvenile_	5.217	2.830	0.383	9.768
γ_0,2013,juvenile_	−5.322	2.121	−10.860	−2.526
σ_2013,juvenile_	3.746	2.084	1.058	8.935
γ_0,2014,juvenile_	−3.170	0.739	−4.874	−1.949
σ_2014,juvenile_	1.696	0.881	0.638	3.958
γ_0,2011,adult_	−3.784	0.408	−4.694	−3.116
σ_2011,adult_	0.504	0.442	0.013	1.610
γ_0,2012,adult_	−4.759	1.043	−7.473	−3.506
σ_2012,adult_	1.570	1.342	0.099	5.304
γ_0,2013,adult_	−5.407	1.354	−8.754	−3.544
σ_2013,adult_	2.416	1.462	0.790	6.632
γ_0,2014,adult_	−4.050	0.480	−5.148	−3.324
σ_2014,adult_	0.669	0.606	0.034	2.148
Deviance	937.677	10.580	918.400	960.000

a Additional model description included in Figure S3.

Model selection results for cause‐specific probabilities including expert knowledge demonstrated no evidence for a difference in years or age groups with the BASELINE model being selected as the most parsimonious (Table 4). In contrast, when we assumed cause‐of‐death was known, model rankings changed. The most parsimonious model, which received no support when including expert knowledge (∆DIC =4.14; Table 4), contained independent annual cause‐specific probabilities (Table 4). The BASELINE model was a competing model (∆DIC =0.17). When we compared parameter estimates from the BASELINE model that did not include expert knowledge to the BASELINE model including observer‐specified priors, as expected, *SD* decreased for intercept parameter offsets of the multinomial probabilities.

**Table 4:** Comparison for cause‐specific probability models for each suite of models, including uncertainty and no uncertainty associated with mortality cause, for Wisconsin white‐tailed deer from 2011 to 2014. All models allowed for different cause‐specific categorical probabilities among nine 4‐week intervals based on an interval regularizing parameter. The BASELINE model only estimates interval‐specific parameters assuming no difference among AGE and YEAR. Subscripts add estimated additive differences among age classes or years and int estimated independent age or year effect

Including uncertainty				No uncertainty			
Model^a^	DIC	pD	Δ DIC	Model^a^	DIC	pD	Δ DIC
BASELINE	237.74	36.10	0.00	YEAR_int_	219.637	29.795	0.00
YEAR_add_	238.74	42.06	0.99	BASELINE	219.807	8.823	0.17
AGE_add_	240.47	37.87	2.73	AGE_add_	222.087	10.611	2.45
YEAR_int_	241.89	74.33	4.14	AGE_int_	223.826	16.315	4.19
AGE_int_	243.77	48.23	6.03	YEAR_add_	226.439	14.625	6.80

a Additional model description included in Figure S3.

**Figure 2:**
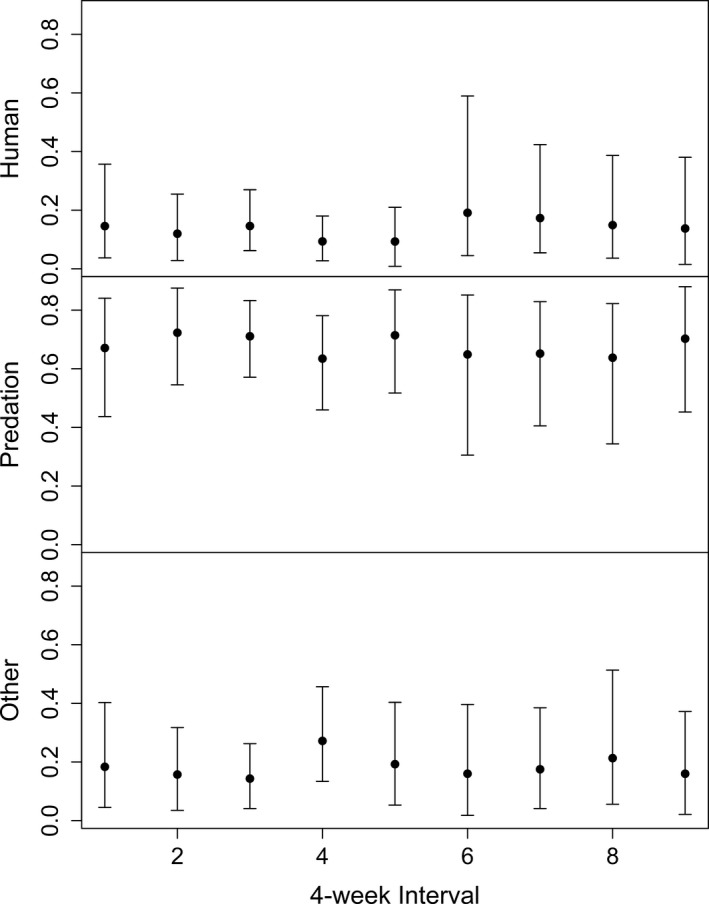
Regularized estimates for cause‐specific categorical probabilities with 95% credible intervals for each 4‐week interval (*N* = 9) outside the hunting season from 2011 to 2014 in Wisconsin, USA, using our BASELINE model and including expert knowledge regarding the uncertainty of the cause‐of‐death assignments

Using the BASELINE model incorporating expert knowledge, estimates of cause‐specific probabilities suggested the highest probability associated with predation followed by other causes‐of‐death and finally human‐associated causes (Table 5, Figure 2). Monthly credible intervals for predation probabilities did not overlap the credible intervals of the other two categories of causes in any month except 4, 6, 7, and 8 (Figure 2). In contrast, when we did not incorporate expert knowledge, differences between conditional probabilities of the three causes‐of‐death occurred only in 2014 (Table 5).

**Table 5:** Comparison of parameter estimates for the model of cause‐specific probabilities for the model including expert knowledge in cause‐of‐death assignments and the model with no uncertainty in these assignments for Wisconsin white‐tailed deer from 2011 to 2014. Both model were regularized for different cause‐specific probabilities among nine monthly intervals, but the model with no expert knowledge included independent year effects: $N(\eta_{0,k,.},\sigma_{k,.}^{2})$. Three causes were modeled, namely human, predation (reference), and all other causes. All parameters are on the log odds scale

Expert knowledge	Parameter^a^	Mean	*SD*	2.50%	97.50%
Yes	η_0,human_	−1.722	0.433	−2.579	−0.851
No	η_0,human,2011_	−0.855	0.704	−2.351	0.488
No	η_0,human,2012_	−1.446	1.073	−3.767	0.560
No	η_0,human,2013_	−1.293	1.401	−4.281	1.283
No	η_0,human,2014_	−2.151	0.967	−4.310	−0.249
Yes	σ_human_	0.682	0.580	0.041	2.168
No	σ_human,2011_	1.381	1.406	0.032	5.367
No	σ_human,2012_	2.353	2.197	0.081	8.416
No	σ_human,2013_	5.479	2.476	1.134	9.731
No	σ_human,2014_	2.295	2.207	0.071	8.367
Yes	η_0,other_	−1.415	0.415	−2.304	−0.662
No	η_0,other,2011_	−1.721	1.195	−4.421	0.334
No	η_0,other,2012_	−1.898	1.344	−4.870	0.519
No	η_0,other,2013_	−0.863	0.982	−3.023	1.101
No	η_0,other,2014_	−1.475	0.489	−2.515	−0.577
Yes	σ_other_	0.649	0.494	0.064	1.880
No	σ_other,2011_	3.136	2.153	0.346	8.719
No	σ_other,2012_	3.312	2.603	0.121	9.276
No	σ_other,2013_	2.722	2.449	0.073	8.921
No	σ_other,2014_	0.639	0.660	0.026	2.330
Yes	Deviance	201.639	8.500	185.500	218.700
No	Deviance	189.842	8.174	174.900	207.000

a Additional model description included in Figure S3.

**Figure 3:**
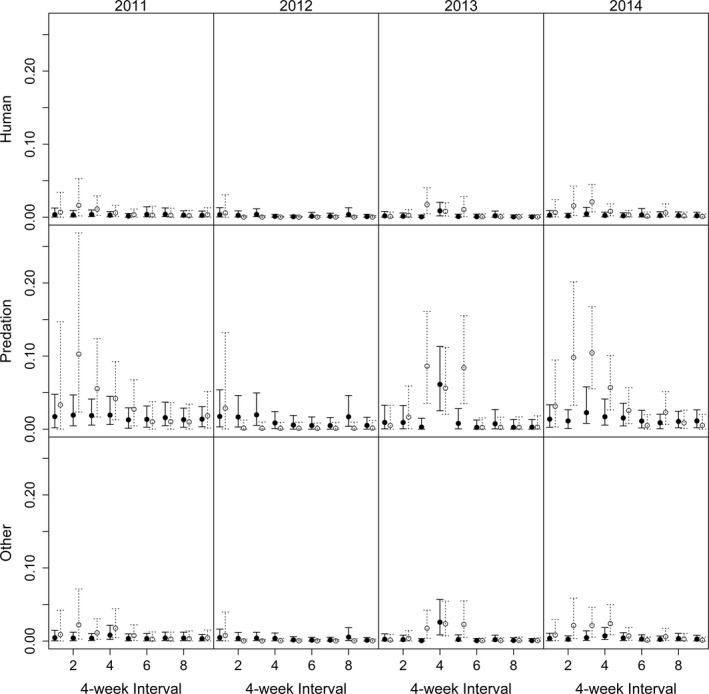
Regularized estimates for overall probability of mortality due to humans, predation, and all other causes with 95% credible intervals for each 4‐week interval (*N* = 9) outside the hunting season for adult (>19 months old; black lines with solid circles) and juvenile (~7–15 months old; dashed gray lines with open circles) white‐tailed deer from 2011 to 2014 in Wisconsin, USA, using a model with independent year and age effects for the overall hazard and the BASELINE model for multinomial logit cause‐specific categorical probabilities

The product of components 1 and 2 of our model incorporating observer prior information illustrates the unconditional probability of dying from each source of mortality (i.e., monthly and age group cause‐specific mortality; Figure 3). Annual predation rates outside the hunting season ranged from 0.038 in 2012 to 0.355 in 2014 for juveniles and 0.098 in 2013 to 0.142 in 2011 for adults. The highest nonpredation annual mortality rate was 0.093 for other in 2014.

## 4 DISCUSSION

### 4.1 Simulations

Our simulations demonstrated that the explicit use of expert knowledge provided a substantial improvement in terms of reduced bias and credible interval coverage of estimated parameters when investigating cause‐specific mortality. Conversely, failing to include this knowledge resulted in large biases and poor coverage of credible intervals of cause‐specific parameter estimates that likely would impact inference. Therefore, when modeling cause‐specific mortality, simulation results support the inclusion of expert knowledge regarding observer uncertainty in cause‐of‐death assignment to improve model performance and ensure rigorous inference.

### 4.2 Applied example

Acknowledging true cause‐of‐death was uncertain in ~45% of observed mortalities clearly impacted inference. When we did not include expert knowledge regarding likelihood of the various causes‐of‐death, we selected a cause‐specific categorical probability model with independent annual estimates and we assumed cause‐specific probabilities varied across years. However, these independent annual estimates were not supported when expert knowledge was included in our model. This difference arose from an inappropriate increase in precision of parameter estimates in the model that did not include expert knowledge. Additionally conditional on dying, probability of mortality due to predation was significantly higher than other causes when including expert knowledge, whereas when uncertainties in cause‐of‐death assignments were ignored, it was only estimated to be higher in the final study year. This has significant implications for conservation and management because misinterpretation of contribution of different sources of mortality can lead to inappropriate and often controversial actions (e.g., predator control). Thus even in this case, where only ~45% of the moralities had some uncertainty associated with their cause, use of expert knowledge was not just an academic exercise, but had direct influence on scientific inference.

There were noticeable differences between the sizes of credible intervals around individual hazard rate estimates between years, particularly for juveniles (Figure 1), which is attributable to a variation in sample sizes and the number of marked deer dying annually. For example, the hazard rates for juveniles in 2012 are noticeably more precise across four‐week intervals when compared to other study years. This is because only one marked juvenile deer died in 2012, which led to low and precise estimates of the hazard rates. Mortality was considerably higher in other years.

The results reported from our model, including expert knowledge, align well with previous research and biological expectations. For example, during the four years of our study, we documented a wide range of mortality patterns among years and age groups (Norton, 2015). Juvenile mortality rates were higher than adult mortality rates outside the hunting season; however, these differences varied within years (Figure 1), possibly related to winter severity (Norton, 2015).

In agreement with others (Gaillard, Festa‐Bianchet, & Yoccoz, 1998; Gaillard, Festa‐Bianchet, Yoccoz, Loison, & Toigo, 2000), there was much less annual variation for adults, and total mortality ranged only 6.7% among all four years. Monthly cumulative hazard predictions for juveniles were usually higher than adult hazards, and they increased earlier and decreased later, through months 4 and 5 (~end of May). This supports other findings that adults are less sensitive to overwinter stress (DelGiudice, Fieberg, Riggs, Carstensen Powell, & Pan, 2006; Gaillard et al., 1998, 2000; Lukacs et al., 2009), and effects of winter may affect juveniles through late winter, even through May in our case, when there was an abundance of nutritious vegetation.

When examining the specific causes of mortality, predation events occurred at the highest probability, similar to McNay and Voller (1995), followed by other, then human events. The difference between predation and nonpredation mortality sources was more apparent in the beginning of the year, than during the winter months.

## 5 CONCLUSION

Our model development overcomes the inability of current techniques to capture additional variability associated with observer uncertainty in cause‐of‐death assignments, and provides a means of incorporating expert knowledge into cause‐specific mortality models in a rigorous manner. The results from our simulation and applied example clearly demonstrate the importance of using expert knowledge explicitly to account for uncertainty of outcomes, not only for proper accounting of variability, but also for drawing appropriate inference from the statistical analyses. Lastly, our modeling approach can be extended to other types of ecological investigations (e.g., dispersal, migration, disease status) where expert knowledge is implicitly used because of uncertainty regarding outcomes, and provides a framework for explicitly integrating expert knowledge into the structure of ecological models.

## CONFLICT OF INTEREST

None declared.

## AUTHOR CONTRIBUTION

AN, DW, DS, TV, and DH developed methodology and ideas; AN, TV, and DS acquired data; DW and AN analyzed the data; DW and AN led the writing of the manuscript. All authors contributed critically to the drafts and gave final approval for publication.

## DATA ACCESSIBILITY

Data used in this study are freely accessible via DRYAD: https://doi.org/10.5061/dryad.v1kq6.
