# Supplementary material for: Using expert knowledge to incorporate uncertainty in cause‐of‐death assignments for modeling of cause‐specific mortality
Source: Ecol Evol. 2017 Nov 30;8(1):509–20. doi: 10.1002/ece3.3701 (PMC5756890; doi:10.1002/ece3.3701)
Supplement: Supplementary file 1 [file ECE3-8-509-s001.pdf]

Supplemental Figures

Fig. S1 - Multinomial Hazard Partitioning Proof

We provide a proof justifying the decomposition of the overall hazard, $h(t)$, into multiple causes using the categorical distribution to estimate cause-specific mortality based on the cumulative incidence function. We begin by defining the following statistics where t = time and T = a random variable of the time of death:

- survival function as $S(t) = exp\left(-\int\limits_0^t h(u)du\right)$
- overall mortality function as $M(t+\Delta|t) = p(t < T < t + \Delta t|T > t) = 1 - S(t+\Delta|t)$
- $h(t) = \lim\limits_{\Delta\to 0} \frac{M(t+\Delta|t)}{\Delta} = \lim\limits_{\Delta\to 0} \frac{p(t<T<t+\Delta|T>t)=1-S(t+\Delta|t)}{\Delta}$
- cumulative incidence function as $CIF = \int\limits_0^t h(u)S(u)du$
- cause-specific mortality for the kth source of mortality as $M_k(t+\Delta|t) = p(t < T < t+\Delta t, K = k|T > t)$
- cause-specific hazard rates as $h_k(t) = \lim\limits_{\Delta\to 0} \frac{M_k(t+\Delta|t)}{\Delta} = \lim\limits_{\Delta\to 0} \frac{p(t<T<t+\Delta,K=k|T>t)}{\Delta}$
- cause-specific cumulative incidence function as $CIF_k = \int\limits_0^t h_k(u)S(u)du$
- the conditional probability the death is due to cause $k = \pi_k = p(K = k|h(t))$.

Finally, let $A = p(t < T < t + \Delta t|T > t)$ and $B_k = p(K = k)$, and thus $h_k(t) = \lim\limits_{\Delta\to 0} \frac{p(A,B_k|T>t)}{\Delta}$ and $h(t) = \lim\limits_{\Delta\to 0} \frac{p(A|T>t)}{\Delta}$.

Proof

Based on the concept of the conservation of the mortality and marginalizing over B:

$$p(A) = \sum_k h_k(t) = \sum_k p(A, B_k).$$

Then the following equality holds true:

$$\frac{h_k(t)}{h(t)} = \frac{p(A, B_k)}{p(A)}.$$

Employing the definition of conditional probability yields:

$$\frac{p(A, B_k)}{p(A)} = p(B_k|A).$$

A simple rearrangement of terms results in:

$$p(A, B_k) = p(B_k|A) \times p(A),$$

and by substitution

$$p(B_k|A) = \pi_k \times h(t).$$

This leads to the desired results:

$$h_k(t) = \lim_{\Delta\to 0} \frac{p(\pi_k \times h(t)|T > t)}{\Delta}.$$

BUGS Code for Estimating Cause-specific Mortality

Fig. S2 - Model - WinBUGS YEARint + AGEadd model for overall cumulative hazard

```
model {
        for (j in 1:N) {

  # Bernoulli trick to estimate mortality probability
         event[j] ~ dbern(UM[interval[j],j])

  # change in variable to mortality probability scale (cloglog link)
         UM[interval[j],j] <- 1-exp(-hazard[interval[j],j])

  # linear model for log cumulative hazard
         hazard[interval[j],j] <- exp(gamma[interval[j],year[j]]+
                        betaadult*adult[j])
        }

        for (i in 1:Nint) {
                for (j in 1:4){

 # interval smoothing for each year
                        gamma[i,j] ~ dnorm(mu[j],tau[j])
                }
         }

        for (j in 1:4){

 # vague prior for intercept grand mean by year
                        mu[j]~dnorm(0,0.0001)

 # vague prior for among interval variation by year
                        sd[j]~dunif(0,10)
                        tau[j] <- 1/(sd[j]*sd[j])
        }

 # vague prior for age group contrast parameter
                betaadult~dnorm(0,0.0001)
}
```

Data definitions:

- adult = indicator for age (0 = juvenile, 1 = adult)
- event = the status of an individual during encounter (0 = no event, 1 = event)
- interval = interval when the encounter occurred (1:9)
- N = total number of encounters for every animal (if available during interval)
- Nint = intervals (9)
- year = year when the encounter occurred (1 = 2011, 2 = 2012, 3 = 2013, 4 = 2014)

Stochastic node definitions:

- betaadult = additive term for age group contrast

- gamma = intercept term for log cumulative hazard
- mu = grand mean intercept for each year
- sd = standard deviation for among interval regularizing parameter by year

Fig. S3 - WinBUGS YEARadd + AGEadd model for cause-specific Multinomial probabilities with uncertainty in event classification for 3 events

```
model {
        for (i in 1:Nint){

# interval smoothing for cause 1
                Alpha[1,i]~dnorm(Alpha1,a1tau)

# reference cause (2) will be smoothed based on logit
## multinomial change in variable
                Alpha[2,i]<-0

# interval smoothing for cause 3
                Alpha[3,i]~dnorm(Alpha3,a3tau)
        }
        for (i in 1:Ncases){

# sums exp(Betas) logit multinomial change in denominator
                TEB[leftfate[i],i]<-sum(EB[1:Ncauses,leftfate[i],i])

# exp(linear model for cause-specific probabilities)
                for (j in 1:Ncauses) {
                        EB[j,leftfate[i],i]<-exp(Alpha[j,leftfate[i]]+
                        Beta12[j]*yr12[i]+ Beta13[j]*yr13[i]+
                        Beta14[j]*yr14[i]+Betaadult[j]*adult[i])

# standard multinomial logistic probability
                        P[j,leftfate[i],i]<-EB[j,leftfate[i],i]/
                                                TEB[leftfate[i],i]

# log of the probability
                LP[j,leftfate[i],i]<-log(P[j,leftfate[i],i])
                }
        }
# Dirichlet priors
        for (i in 1:K) {

# generates numerator values from gamma for Dirichlet prior
                g[i]~dgamma(alpha[i],alpha[i])
        }

# sums numerator values for standardizing denominator
## for Dirichlet prior from gamma
        totg<-sum(g[1:K])

        for (i in 1:K) {

# probability for each cause for Dirichlet prior
                p.int[i] <- g[i]/totg
        }

# standardized scaled probability for cause 1 for Dirichlet prior
        scalep.int[1] <- p.int[1]/p.int[2]
```

```
# log scaled probability for cause 1 grand mean intercept prior
        Alpha1<- log(scalep.int[1])

# standardized scaled probability for cause 3 for Dirichlet prior
        scalep.int[3] <- p.int[3]/p.int[2]

# log scaled probability for cause 3 grand mean intercept prior
                ##(as a function of other causes)
        Alpha3<- log(scalep.int[3])

# vague prior for 2012 versus 2011 for cause 1
        Beta12[1]~dnorm(0,0.001)

# reference cause (2) for 2012 versus 2011 contrast
        Beta12[2]<-0

# vague prior for 2012 versus 2011 for cause 3
        Beta12[3]~dnorm(0,0.001)

# vague prior for 2013 versus 2011 for cause 1
        Beta13[1]~dnorm(0,0.001)

# reference cause (2) for 2013 versus 2011 contrast
        Beta13[2]<-0

# vague prior for 2013 versus 2011 for cause 3
        Beta13[3]~dnorm(0,0.001)

# vague prior for 2014 versus 2011 for cause 1
        Beta14[1]~dnorm(0,0.001)

# reference cause (2) for 2014 versus 2011 contrast
        Beta14[2]<-0

# vague prior for 2014 versus 2011 for cause 3
        Beta14[3]~dnorm(0,0.001)

# vague prior for age group contrast for cause 1
        Betaadult[1]~dnorm(0,0.001)

# reference cause (2) for age group contrast
        Betaadult[2]<-0

# vague prior for age group contrast for cause 3
        Betaadult[3]~dnorm(0,0.001)

# vague prior for cause 1 among interval variation (cause 2 is reference)
        a1sd~dunif(0,10)
        a1tau <- 1/(a1sd*a1sd)

# vague prior for cause 3 among interval variation (cause 2 is reference)
        a3sd~dunif(0,10)
        a3tau <- 1/(a3sd*a3sd)
```

```
# generates a hypothetical true observation from predictive distribution
        for (case in 1:Ncases) {

# picks off the corresponding log likelihood contribution, converts
##to likelihood
                Yaug[leftfate[case],case,1:Ncauses]~dmulti(PV[case,1:Ncauses],1)

#Ones trick - accumulates the likelihood product over all Ncases:
            Plike[case]<-exp(Yaug[leftfate[case],case,1]*LP[1,leftfate[case],case]+
                Yaug[leftfate[case],case,2]*LP[2,leftfate[case],case]+
                Yaug[leftfate[case],case,3]*LP[3,leftfate[case],case])

#Ones trick to allow WinBUGS to augment causes
##from prior prediction probabilities (PP)
                Ones[case]~dbern(Plike[case])
        }
}
```

Data definitions:

- adult = indicator for age (0 = juvenile, 1 = adult)
- alpha = prior for Dirichlet distribution using Gamma distribution trick (See WinBUGS User Manual "Learning about the parameters of a Dirichlet distribution)
- K = number of causes
- leftfate = interval when event occurred
- Ncases = total number of events
- Nint = intervals (9)
- Ones = vector of 1's of 'Ncases' length for the 'Ones trick'
- PV = vector of prior prediction probabilities associated with each cause for each event
- yr12 = indicator for year 2012
- yr13 = indicator for year 2013
- yr14 = indicator for year 2014

Stochastic node definitions:

- Alpha = intercept term for logit cause-specific multinomial probability
- a1sd = standard deviation for among interval variation for cause 1
- a3sd = standard deviation for among interval variation for cause 3
- Betaadult = additive term for age group contrast for each cause
- Beta12 = additive term for 2012 versus 2011 contrast for each cause
- Beta13 = additive term for 2013 versus 2011 contrast for each cause
- Beta14 = additive term for 2014 versus 2011 contrast for each cause
- g = used to construct prior based on Dirichlet using Gamma distribution
- Yaug = vector augmented data for cause (1's or 0's) based on prior prediction probabilities

Derived node definitions:

- Alpha1 = grand mean for intercept term for logit cause-specific multinomial probability for cause 1
- Alpha3 = grand mean for intercept term for logit cause-specific multinomial probability for cause 3
- a1tau = precision for among interval regularizing parameter for cause 1
- a3tau = precision for among interval regularizing parameter for cause 3
- EB = cause-specific numerator for multinomial logit link
- LP = log multinomial cause-specific probability
- P = multinomial cause-specific probability
- pint = cause-specific numerator for constructing Dirichlet prior from Gamma distribution
- Plike = augmented event from predicted values (PV), updated for each MCMC iteration
- scalep.int = e scale prior probability for Dirichlet distribution
- TEB = standardizing denominator for multinomial logit link
- totg = standardizing denominator for constructing Dirichlet prior from Gamma distribution

The following figures depict the results of simulations of 500 datasets evaluating the impacts of including uncertainty in the classification of the sources of mortality via an observer specified vector of prior predictive values for each potential source of mortality (prior model) compared to the model where observers do not include their uncertainty regarding the assigned cause-of-death (no prior model) and to the model where there is no error in observer assignments (no error model). The simulations were run over a variety of underlying probabilities of death (i.e., prevalences) due to 4 potential sources of mortalities and a range of misclassification rates.

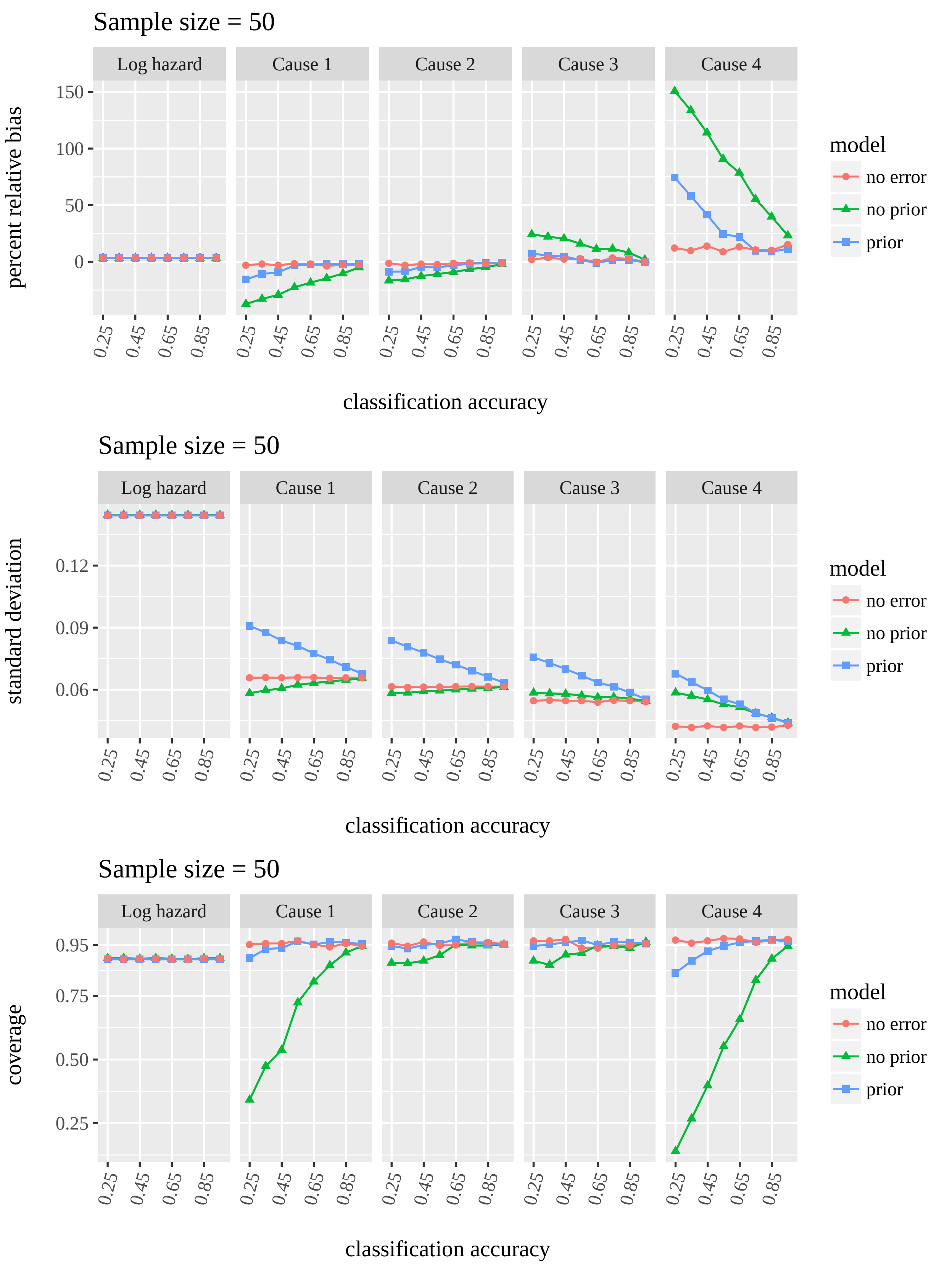

Fig. 4: Simulation results for 4 sources of mortality where the true probability of dying from each source is 0.4, 0.3, 0.2 and 0.1, and the sample size was 50 marked individuals.

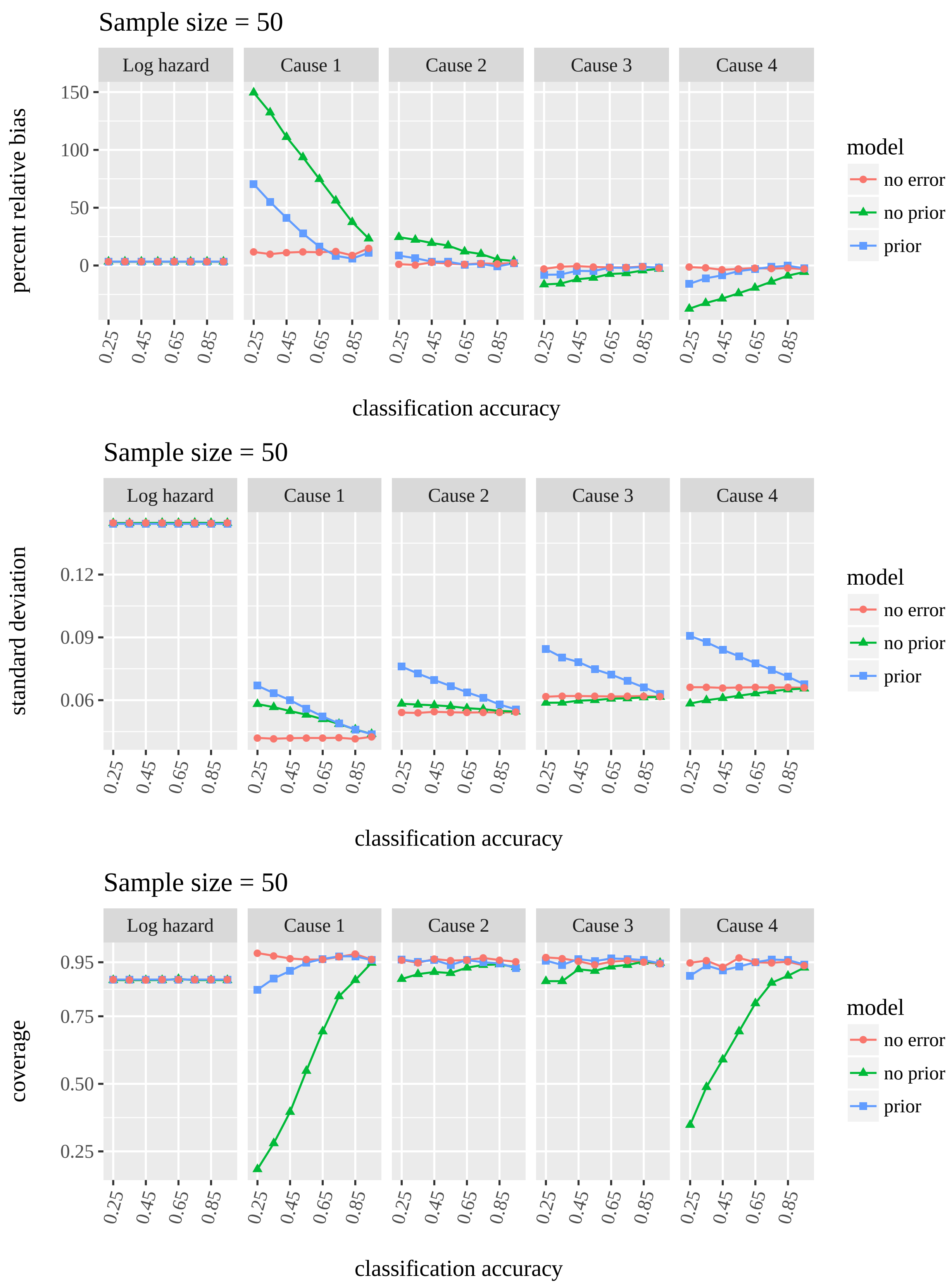

Fig. 5: Simulation results for 4 sources of mortality where the true probability of dying from each source is 0.1, 0.2, 0.3 and 0.4, and the sample size was 50 marked individuals.

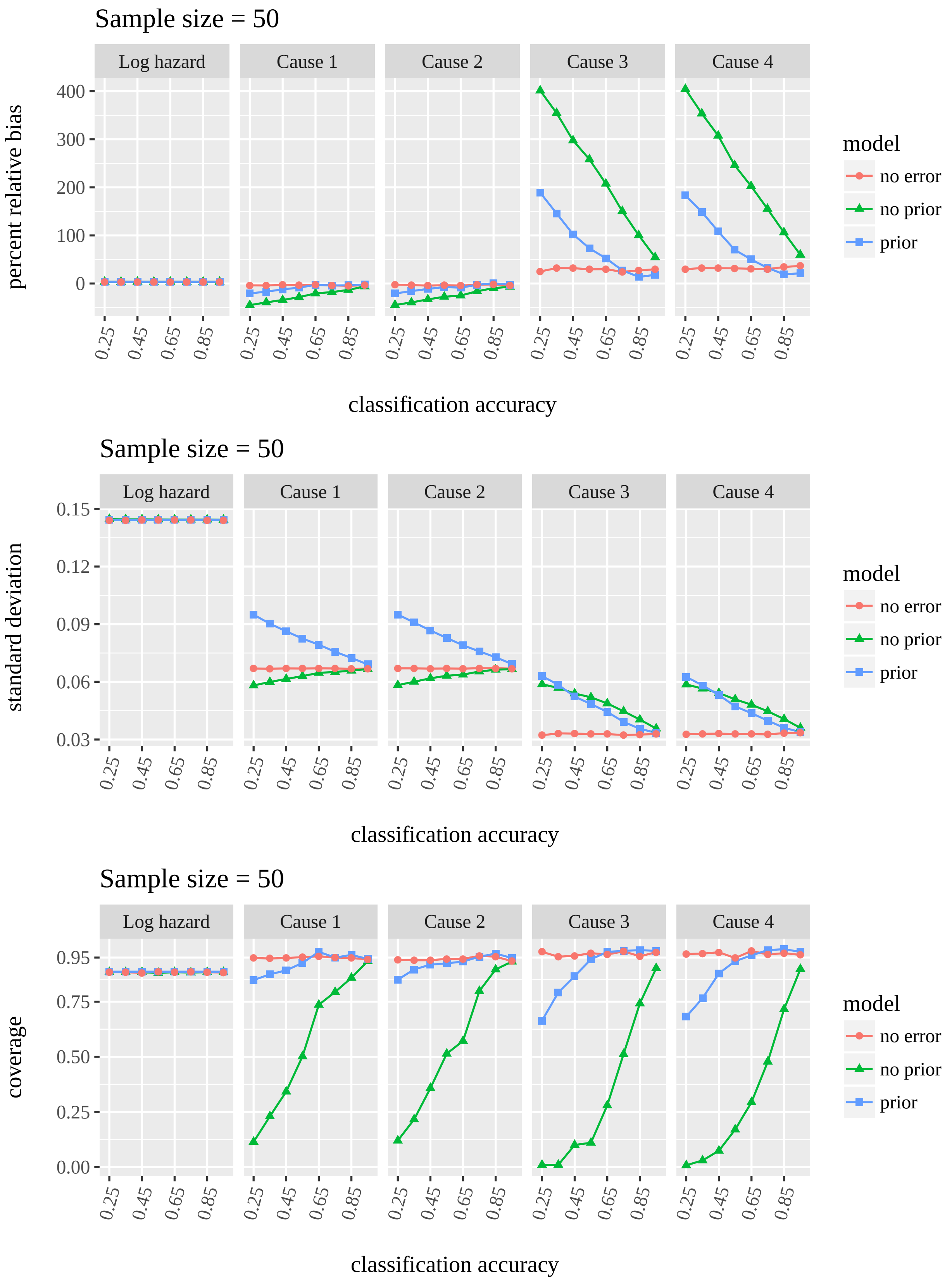

Fig. 6: Simulation results for 4 sources of mortality where the true probability of dying from each source is 0.45, 0.45, 0.05 and 0.05, and the sample size was 50 marked individuals.

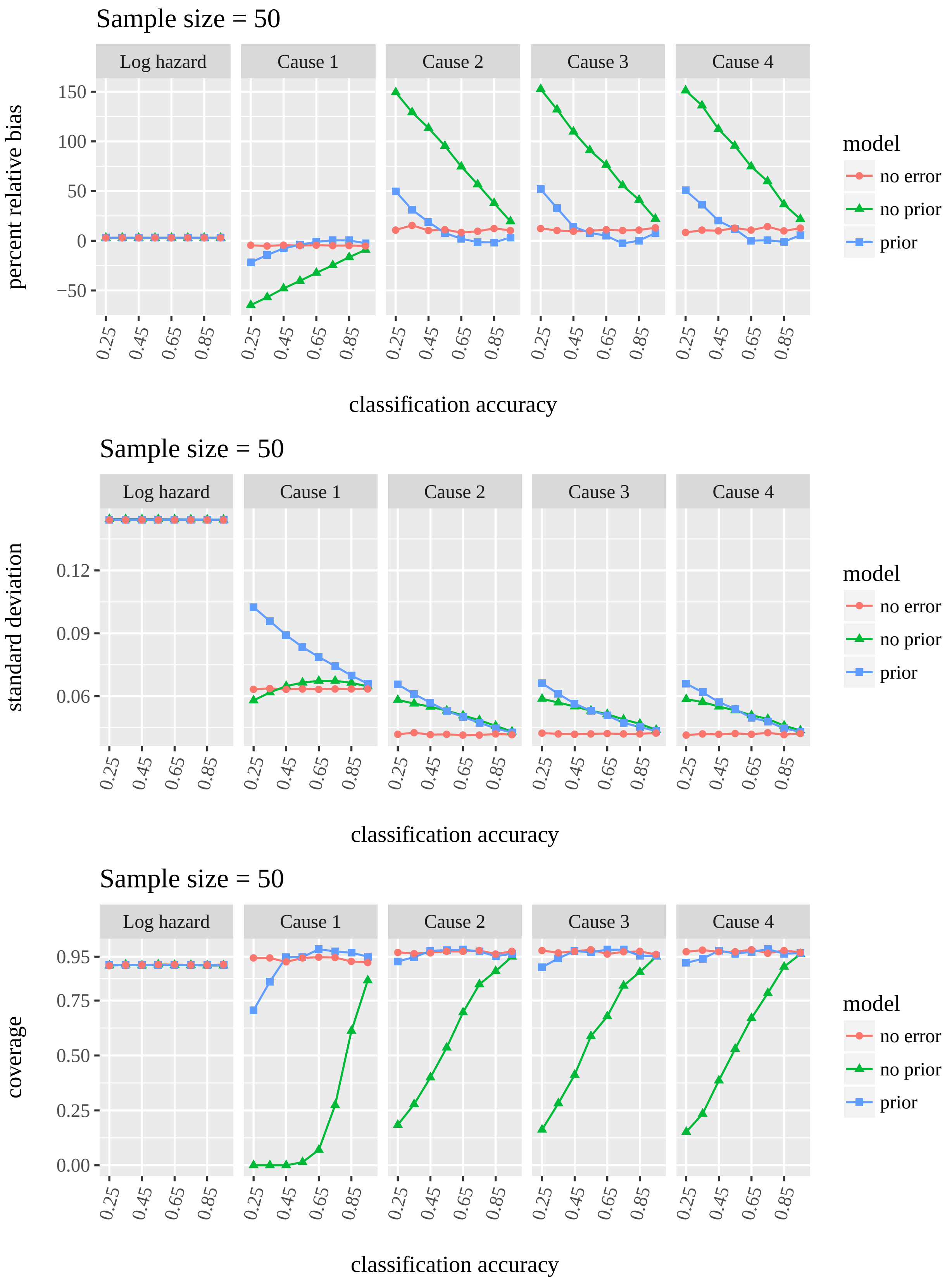

Fig. 7: Simulation results for 4 sources of mortality where the true probability of dying from each source is 0.7, 0.1, 0.1 and 0.1, and the sample size was 50 marked individuals.

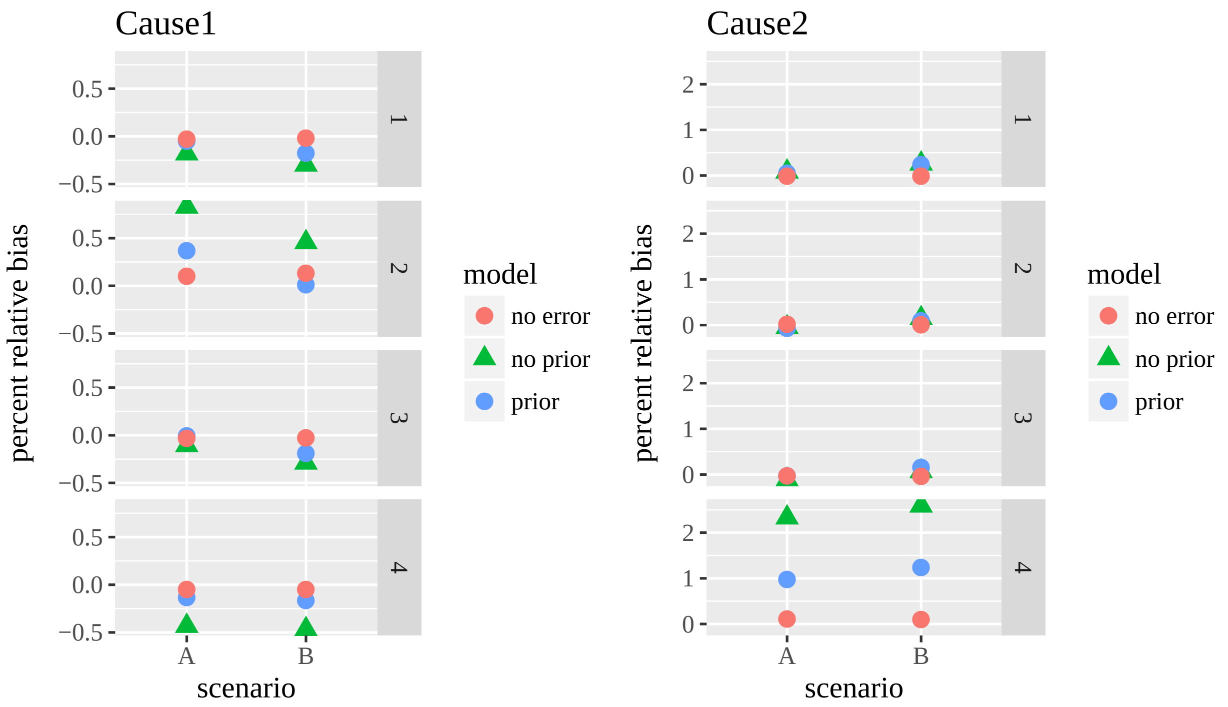

Fig. 8: Simulation results depicting the difference in percent relative bias of the first two parameters between the “no prior”, “prior”, and ”no error in assignment” models for 4 sources of mortality where the true probability of dying from each source was 0.4, 0.3, 0.2, 0.1 for case 1, 0.1, 0.2, 0.3, 0.4 for case 2, 0.45, 0.45, 0.05, 0.05 for case 3, and 0.70, 0.1, 0.1, 0.1 for case 4. Scenario A are results from the following classification matrix: $\begin{bmatrix} 0.5 & 0.4 & 0.05 & 0.05 \\ 0.4 & 0.5 & 0.05 & 0.05 \\ 0.07 & 0.07 & 0.8 & 0.07 \\ 0.07 & 0.07 & 0.07 & 0.8 \end{bmatrix}$. Scenario B utilized the following classification matrix: $\begin{bmatrix} 0.5 & 0.4 & 0.05 & 0.05 \\ 0.2 & 0.7 & 0.05 & 0.05 \\ 0.07 & 0.07 & 0.8 & 0.07 \\ 0.07 & 0.07 & 0.07 & 0.8 \end{bmatrix}$.

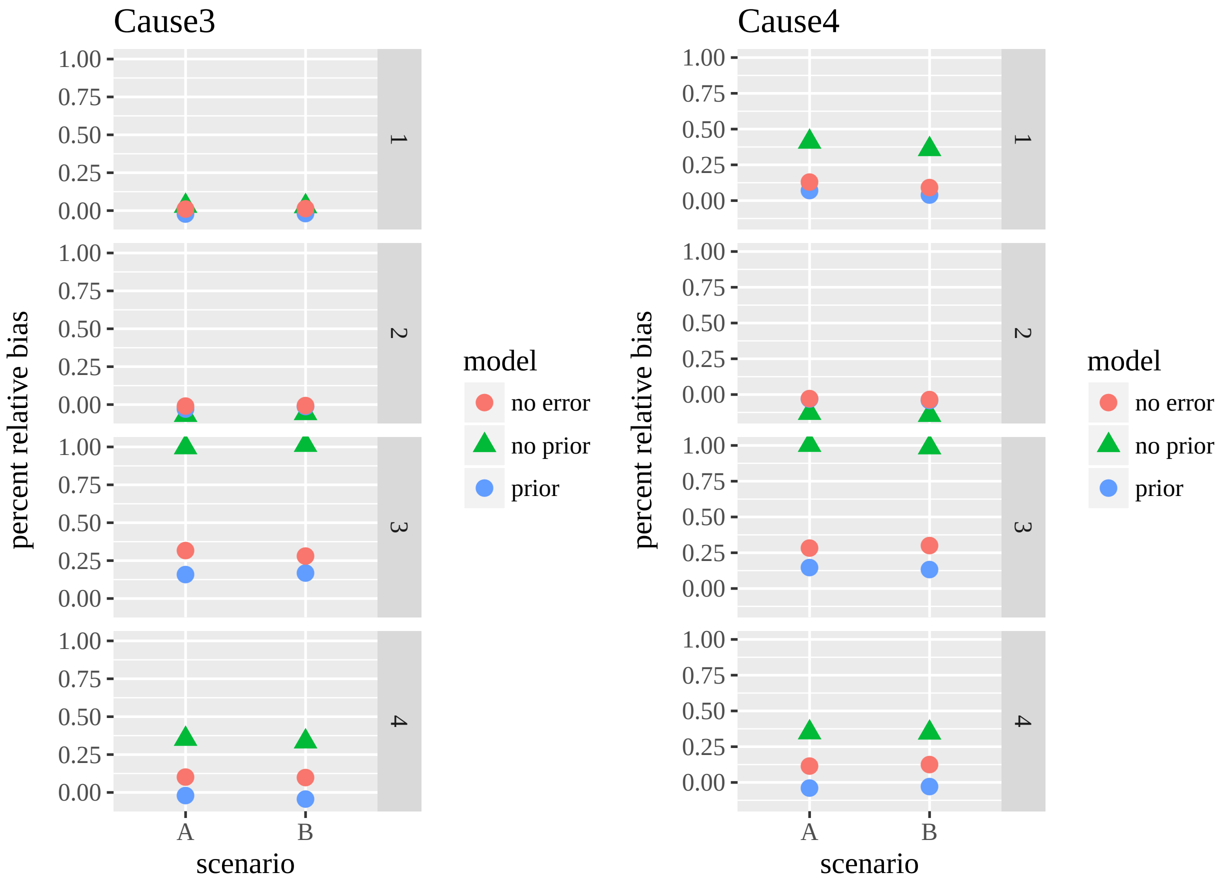

Fig. 9: Simulation results depicting the difference in percent relative bias of the last two parameters between the “no prior”, “prior”, and ”no error in assignment” models for 4 sources of mortality where the true probability of dying from each source was 0.4, 0.3, 0.2, 0.1 ffor case 1, 0.1, 0.2, 0.3, 0.4 for case 2, 0.45, 0.45, 0.05, 0.05 for case 3, and 0.70, 0.1, 0.1, 0.1 for case 4. Scenario A are results from the following classification matrix: $\begin{bmatrix} 0.5 & 0.4 & 0.05 & 0.05 \\ 0.4 & 0.5 & 0.05 & 0.05 \\ 0.07 & 0.07 & 0.8 & 0.07 \\ 0.07 & 0.07 & 0.07 & 0.8 \end{bmatrix}$. Scenario B utilized the following classification matrix: $\begin{bmatrix} 0.5 & 0.4 & 0.05 & 0.05 \\ 0.2 & 0.7 & 0.05 & 0.05 \\ 0.07 & 0.07 & 0.8 & 0.07 \\ 0.07 & 0.07 & 0.07 & 0.8 \end{bmatrix}$.

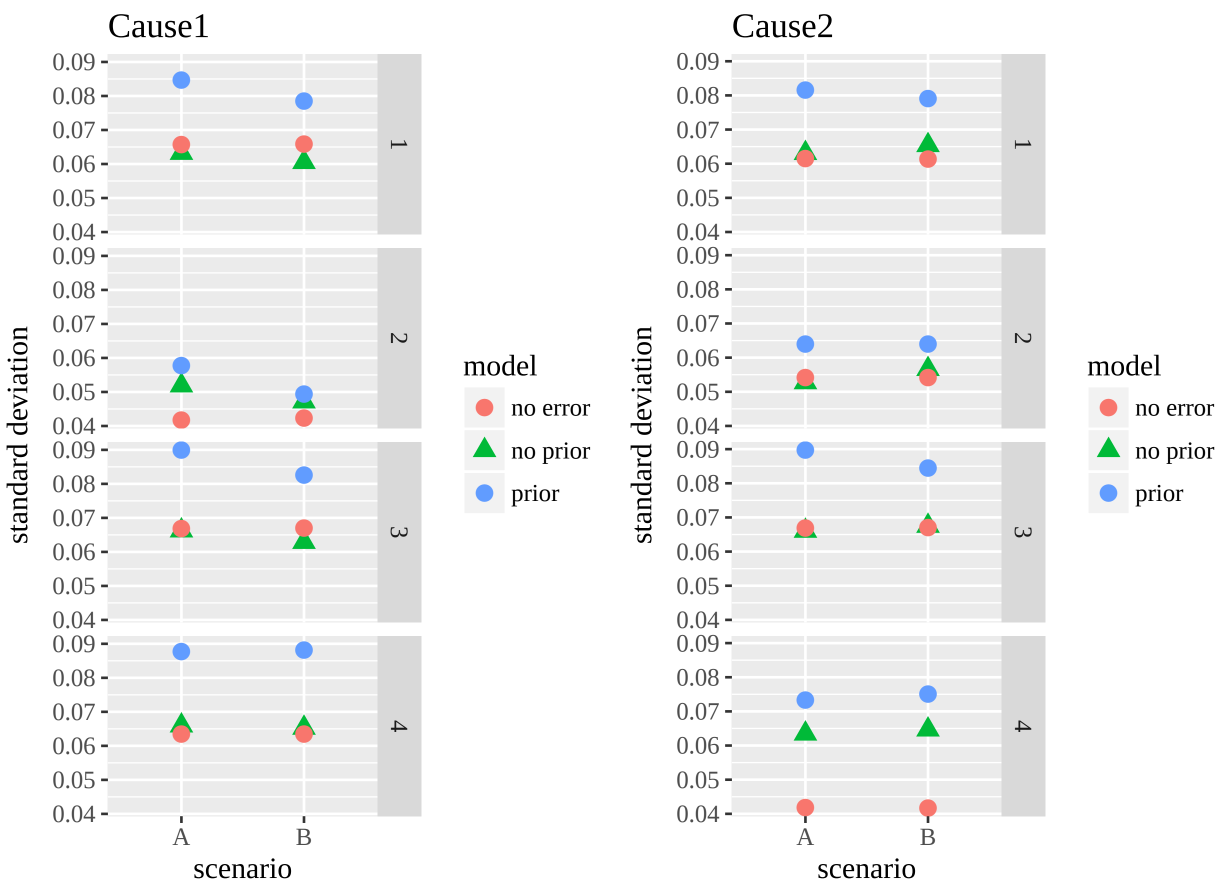

Fig. 10: Simulation results depicting the standard deviations of the first two parameters between the "no prior", "prior", and "no error in assignment" models for 4 sources of mortality where the true probability of dying from each source was 0.4, 0.3, 0.2, 0.1 for case 1, 0.1, 0.2, 0.3, 0.4 for case 2, 0.45, 0.45, 0.05, 0.05 for case 3, and 0.70, 0.1, 0.1, 0.1 for case 4. Scenario A are results from the following classification matrix: $\begin{bmatrix} 0.5 & 0.4 & 0.05 & 0.05 \\ 0.4 & 0.5 & 0.05 & 0.05 \\ 0.07 & 0.07 & 0.8 & 0.07 \\ 0.07 & 0.07 & 0.07 & 0.8 \end{bmatrix}$. Scenario B utilized the following classification matrix: $\begin{bmatrix} 0.5 & 0.4 & 0.05 & 0.05 \\ 0.2 & 0.7 & 0.05 & 0.05 \\ 0.07 & 0.07 & 0.8 & 0.07 \\ 0.07 & 0.07 & 0.07 & 0.8 \end{bmatrix}$.

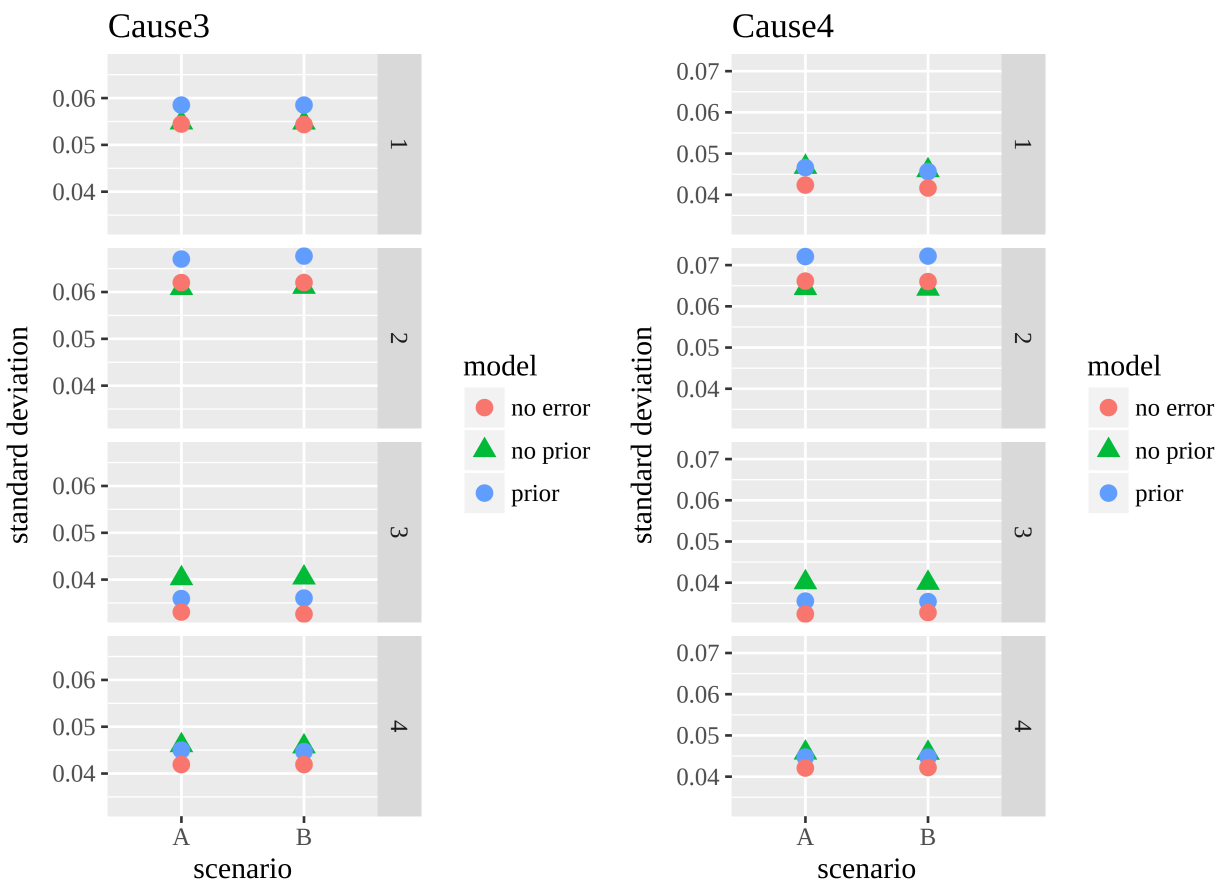

Fig. 11: Simulation results depicting the standard deviations of the last two parameters between the "no prior", "prior", and "no error in assignment" models for 4 sources of mortality where the true probability of dying from each source was 0.4, 0.3, 0.2, 0.1 for case 1, 0.1, 0.2, 0.3, 0.4 for case 2, 0.45, 0.45, 0.05, 0.05 for case 3, and 0.70, 0.1, 0.1, 0.1 for case 4. Scenario A are results from the following classification matrix: $\begin{bmatrix} 0.5 & 0.4 & 0.05 & 0.05 \\ 0.4 & 0.5 & 0.05 & 0.05 \\ 0.07 & 0.07 & 0.8 & 0.07 \\ 0.07 & 0.07 & 0.07 & 0.8 \end{bmatrix}$. Scenario B utilized the following classification matrix: $\begin{bmatrix} 0.5 & 0.4 & 0.05 & 0.05 \\ 0.2 & 0.7 & 0.05 & 0.05 \\ 0.07 & 0.07 & 0.8 & 0.07 \\ 0.07 & 0.07 & 0.07 & 0.8 \end{bmatrix}$.

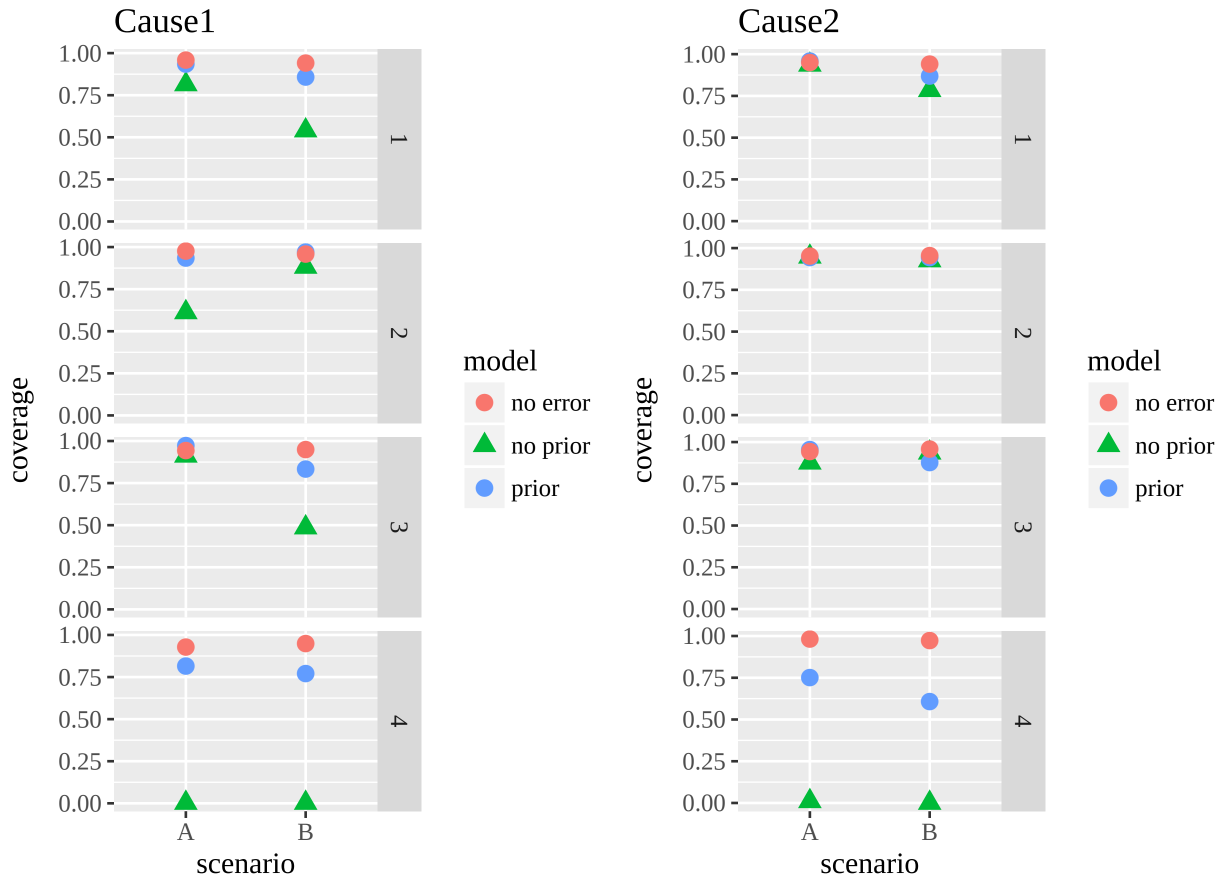

Fig. 12: Simulation results depicting the credible interval coverage of the first two parameters between the “no prior”, “prior”, and ”no error in assignment” models for 4 sources of mortality where the true probability of dying from each source was 0.4, 0.3, 0.2, 0.1 for case 1, 0.1, 0.2, 0.3, 0.4 for case 2, 0.45, 0.45, 0.05, 0.05 for case 3, and 0.70, 0.1, 0.1, 0.1 for case 4. Scenario A are results from the following classification matrix: $\begin{bmatrix} 0.5 & 0.4 & 0.05 & 0.05 \\ 0.4 & 0.5 & 0.05 & 0.05 \\ 0.07 & 0.07 & 0.8 & 0.07 \\ 0.07 & 0.07 & 0.07 & 0.8 \end{bmatrix}$. Scenario B utilized the following classification matrix: $\begin{bmatrix} 0.5 & 0.4 & 0.05 & 0.05 \\ 0.2 & 0.7 & 0.05 & 0.05 \\ 0.07 & 0.07 & 0.8 & 0.07 \\ 0.07 & 0.07 & 0.07 & 0.8 \end{bmatrix}$.

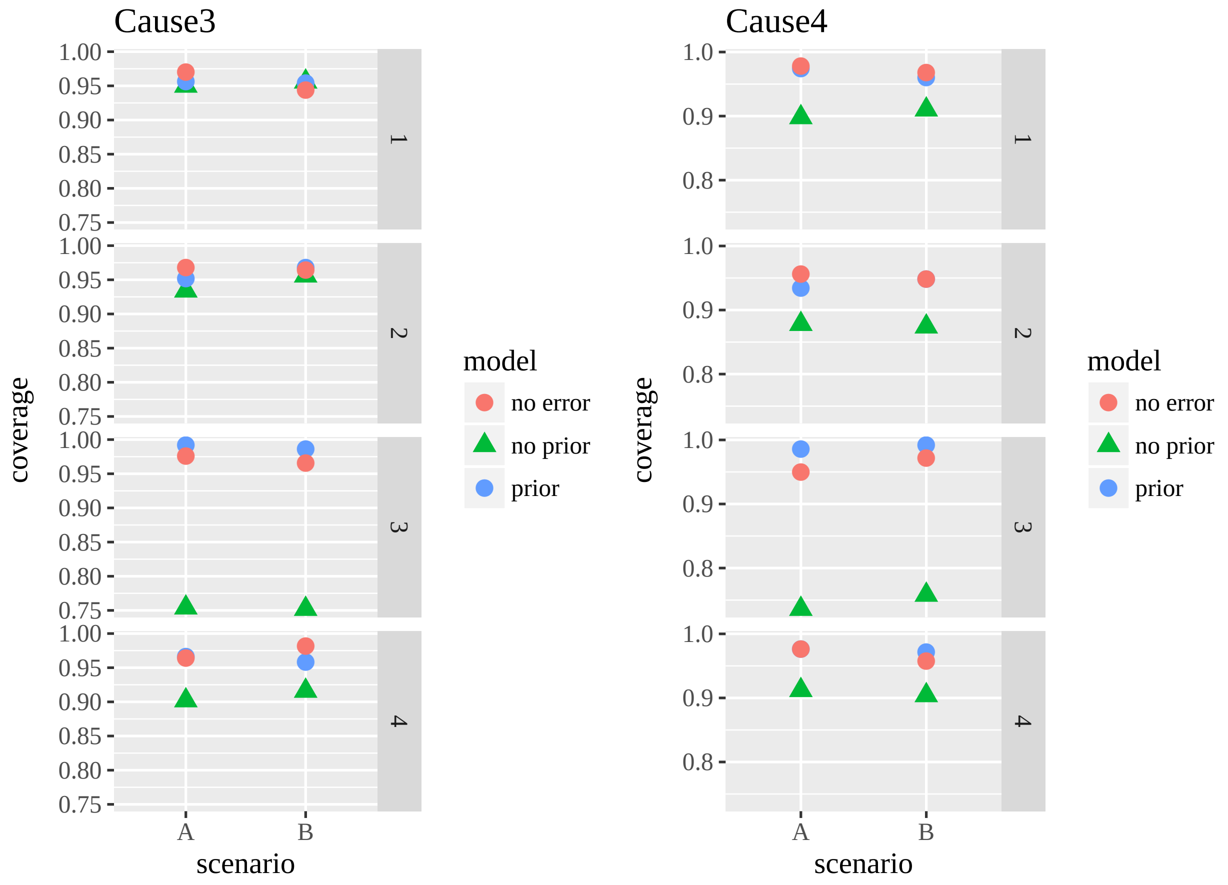

Fig. 13: Simulation results depicting the credible interval coverage of the last two parameters between the "no prior", "prior", and "no error in assignment" models for 4 sources of mortality where the true probability of dying from each source was 0.4, 0.3, 0.2, 0.1 for case 1, 0.1, 0.2, 0.3, 0.4 for case 2, 0.45, 0.45, 0.05, 0.05 for case 3, and 0.70, 0.1, 0.1, 0.1 for case 4. Scenario A are results from the following classification matrix: $\begin{bmatrix} 0.5 & 0.4 & 0.05 & 0.05 \\ 0.4 & 0.5 & 0.05 & 0.05 \\ 0.07 & 0.07 & 0.8 & 0.07 \\ 0.07 & 0.07 & 0.07 & 0.8 \end{bmatrix}$. Scenario B utilized the following classification matrix: $\begin{bmatrix} 0.5 & 0.4 & 0.05 & 0.05 \\ 0.2 & 0.7 & 0.05 & 0.05 \\ 0.07 & 0.07 & 0.8 & 0.07 \\ 0.07 & 0.07 & 0.07 & 0.8 \end{bmatrix}$.

Code S1. R Code Description for Conducting Simulations

Purpose

The purpose of these simulations was to evaluate the effects on estimation of cause-specific hazards of including observer specified priors for each source of morality. These priors were probabilities assigned to each source of mortality, and indicated the observer's latent belief that each source of mortality was the proximal cause of death. We were specifically interested in comparing the bias, variance and credible interval coverage between estimation procedures that included this observer prior information and those that treated the observer assigned cause of death as measured without error. In our simulations we will assume that there are 4 different causes of mortality and observers will be assigning each dead animal to one of those causes.

R Code

The beginning of the R script loads the necessary R libraries.

```
library(R2jags)
library(ggmcmc)
library(matrixStats)
library(coda)
library(knitr)
library(MCMCpack)
library(truncnorm)
library(Rcpp)
library(RcppArmadillo)
library(parallel)

cores<-detectCores()
```

The next section of code creates various functions that will be used during the simulation process. The first function creates confusion matrices (Ψ). These matrices determine the underlying probability of assigning a cause of death to the various sources of mortality conditional on the true cause of death, $Pr\left(E_i = j|T_i = k\right) = \phi_{jk}$, where E_i represents the evidence at a mortality site that is summarized as an error-prone cause of death assigned by the ith mortality event, and T_i is the true underlying cause of death . The columns indices of Ψ represent the true cause of death and the row indices represent the assigned cause of death:

$$\Psi = \begin{bmatrix} \phi_{11} & \phi_{12} & \phi_{13} & \phi_{14} \\ \phi_{21} & \phi_{22} & \phi_{23} & \phi_{24} \\ \phi_{31} & \phi_{32} & \phi_{33} & \phi_{34} \\ \phi_{41} & \phi_{42} & \phi_{43} & \phi_{44} \end{bmatrix}.$$

The diagonal of Ψ represents the probability of correctly assigning cause of death. For simplicity, this function creates the matrices such that the off-diagonal terms are all equal. We also create two additional confusion matrices (classm10 and classm11). The first has large uncertainty separating two of the potential causes of death from each other during assignment (i.e., the two sources of morality are often confused by observers), and the second has large uncertainty separating the first cause of death from the second (i.e., the first source of mortality is often confused with the second by observers, but the converse is not true). Note the columns of the confusion matrices are constrained to sum to 1.

```
###Create classification matrices - various certainties
create.classm<-function(x,dim1){
```

```r
  #equal prob.of incorrect classification
  classm<-matrix((1-x)/(dim1-1),dim1,dim1)

  #correct classification
  diag(classm)<-rep(x,dim1)
  return(classm)
}

classm1<-create.classm(0.25,4)
classm2<-create.classm(0.35,4)
classm3<-create.classm(0.45,4)
classm4<-create.classm(0.55,4)
classm5<-create.classm(0.65,4)
classm6<-create.classm(0.75,4)
classm7<-create.classm(0.85,4)
classm8<-create.classm(0.95,4)
classm9<-create.classm(1,4)

classm10<-matrix(c(0.5,0.4,0.2/3,0.2/3,0.4,0.5,0.2/3,0.2/3,
                   0.05,0.05,0.8,0.2/3,0.05,0.05,0.2/3,0.8),4,4,byrow=T)
classm11<-matrix(c(0.5,0.4,0.05,0.05,0.2,0.7,0.05,0.05,0.2/3,0.2/3,
                   0.8,0.2/3,0.2/3,0.2/3,0.2/3,0.8),4,4)
```

The resulting classification matrices are shown below:

```
## [1] "Classification Matrix 1"
##      [,1] [,2] [,3] [,4]
## [1,] 0.25 0.25 0.25 0.25
## [2,] 0.25 0.25 0.25 0.25
## [3,] 0.25 0.25 0.25 0.25
## [4,] 0.25 0.25 0.25 0.25
## [1] "Classification Matrix 2"
##      [,1] [,2] [,3] [,4]
## [1,] 0.35 0.22 0.22 0.22
## [2,] 0.22 0.35 0.22 0.22
## [3,] 0.22 0.22 0.35 0.22
## [4,] 0.22 0.22 0.22 0.35
## [1] "Classification Matrix 3"
##      [,1] [,2] [,3] [,4]
## [1,] 0.45 0.18 0.18 0.18
## [2,] 0.18 0.45 0.18 0.18
## [3,] 0.18 0.18 0.45 0.18
## [4,] 0.18 0.18 0.18 0.45
## [1] "Classification Matrix 4"
##      [,1] [,2] [,3] [,4]
## [1,] 0.55 0.15 0.15 0.15
## [2,] 0.15 0.55 0.15 0.15
## [3,] 0.15 0.15 0.55 0.15
## [4,] 0.15 0.15 0.15 0.55
## [1] "Classification Matrix 5"
##      [,1] [,2] [,3] [,4]
## [1,] 0.65 0.12 0.12 0.12
## [2,] 0.12 0.65 0.12 0.12
```

```
## [3,] 0.12 0.12 0.65 0.12
## [4,] 0.12 0.12 0.12 0.65
## [1] "Classification Matrix 6"
##      [,1] [,2] [,3] [,4]
## [1,] 0.75 0.08 0.08 0.08
## [2,] 0.08 0.75 0.08 0.08
## [3,] 0.08 0.08 0.75 0.08
## [4,] 0.08 0.08 0.08 0.75
## [1] "Classification Matrix 7"
##      [,1] [,2] [,3] [,4]
## [1,] 0.85 0.05 0.05 0.05
## [2,] 0.05 0.85 0.05 0.05
## [3,] 0.05 0.05 0.85 0.05
## [4,] 0.05 0.05 0.05 0.85
## [1] "Classification Matrix 8"
##      [,1] [,2] [,3] [,4]
## [1,] 0.95 0.02 0.02 0.02
## [2,] 0.02 0.95 0.02 0.02
## [3,] 0.02 0.02 0.95 0.02
## [4,] 0.02 0.02 0.02 0.95
## [1] "Classification Matrix 9"
##      [,1] [,2] [,3] [,4]
## [1,]    1    0    0    0
## [2,]    0    1    0    0
## [3,]    0    0    1    0
## [4,]    0    0    0    1
## [1] "Classification Matrix 10"
##      [,1] [,2] [,3] [,4]
## [1,] 0.50 0.40 0.07 0.07
## [2,] 0.40 0.50 0.07 0.07
## [3,] 0.05 0.05 0.80 0.07
## [4,] 0.05 0.05 0.07 0.80
## [1] "Classification Matrix 11"
##      [,1] [,2] [,3] [,4]
## [1,] 0.50 0.20 0.07 0.07
## [2,] 0.40 0.70 0.07 0.07
## [3,] 0.05 0.05 0.80 0.07
## [4,] 0.05 0.05 0.07 0.80
```

The second function creates the observer specified probabilities (PP) of the likelihood of each cause of death. These probabilities are observer priors for the probability that the true cause of death was due to cause k given the biologist summarized the evidence at a mortality site and assigned the mortality as due to cause j, $Pr\left(T_i = k|E_i = j\right)$. The PPs then correspond to priors on the joint probabilities in the jth row of the Γ matrix where each element is $Pr\left(E_i = j, T_i = k\right)$:

$$\mathit{\Gamma} = \begin{bmatrix} \pi_1\phi_{11} & \pi_2\phi_{12} & \pi_3\phi_{13} & \pi_4\phi_{14} \\ \pi_1\phi_{21} & \pi_2\phi_{22} & \pi_3\phi_{23} & \pi_4\phi_{24} \\ \pi_1\phi_{31} & \pi_2\phi_{32} & \pi_3\phi_{33} & \pi_4\phi_{34} \\ \pi_1\phi_{41} & \pi_2\phi_{42} & \pi_3\phi_{43} & \pi_4\phi_{44} \end{bmatrix},$$

where π_k is the true probability of dying from cause k. This function generates the PPs by accepting as arguments the biologist's assignment j and the jth row of Γ. The PP with the largest probability must be the j^{th}element of the vector of PPs because j was considered the most likely cause of death. Therefore, the function generates the j^{th}element of the PP vector from a constrained Dirichlet distribution with the

parameter $\alpha_j = \pi_j \phi_{jj}$. The function uses a naive rejection sampler to draw a vector of observations from the Dirichlet distribution whose maximum element is constrained to corresponds to the biologist's assignment.

```
gen.PP2<-function(probs,j){
  out<-rep(NA,length(probs))
  temp.results<-rdirichlet(10000,probs)
  temp.trunc<-temp.results[apply(temp.results,1,  #truncate so j is max prob.
                                  function(x){max(x)==x[j]}),]
  out<-temp.trunc[1,]
}
```

The third function determines the biologists assignment of the most likely cause of death based on the body of evidence at the mortality. This is accomplished by randomly drawing an assigned cause using a vector of sampling probabilities whose j^{th} element is:

$$Pr(E_i = j) = \frac{\sum_k \pi_k \phi_{jk}}{\sum_k \pi_k \sum_l \phi_{lk}}.$$

Once the assigned cause of death is determined. The PPs are generated using the function described above. Finally, the true cause of death is assigned by randomly drawing a cause using the following sampling probabilities where the k^{th}element is:

$$Pr(T_i = k | E_i = j) = \frac{\pi_k \phi_{jk}}{\sum_k \pi_k \phi_{jk}}.$$

The associated R code is:

```
rassign<-function(classx,censor1,pis){
  #classx = classification probs
  #censor = indicator of not rt censored
  #pis = probabilities for true cause of death
  #draws sample from pr(true cause, biologist assignment)
  ##conditional on assignment draws order of likelihood of each cause
  ##based on biologists prior on posterior predictive values...not joint parms

  #number of individuals not rt. censored (i.e., die)
  c.records<-sum(censor1==0)

  #create container objects
  prior.assign<-matrix(0,c.records,nrow(classx))
  tc<-ran.assign.call<-rep(0,c.records)

  #create joint prob matrix - prob cause * row of classification matrices
  ##implies rows = biologists assignment, cols= true cause of death
  joint.matrix<-matrix(pis,nrow(classx),ncol(classx),byrow=T)*classx

  #approximate parameters for beta distribution for generating PPs
  joint.matrix2<-joint.matrix/rowSums(joint.matrix)


  #determine biologists assignment probs to each cause
  prob.call<-rowSums(joint.matrix)/sum(joint.matrix)
```

```
  for(i in 1:c.records){

    #biologist actual assignment
    ran.assign.call[i]<-sample(1:ncol(classx),1,prob=prob.call)

    #biologists priors on "positive predictive values"
    # prior.assign[i,]<-gen.PP(joint.matrix2[ran.assign.call[i],],ran.assign.call[i],
    #              c(parms.in$alpha[ran.assign.call[i],ran.assign.call[i]],
    #                parms.in$beta[ran.assign.call[i],ran.assign.call[i]]))
    prior.assign[i,]<-gen.PP2(joint.matrix2[ran.assign.call[i],],ran.assign.call[i])

    #true cause
    tc[i]<-sample(c(1:ncol(classx)),1,prob=joint.matrix[ran.assign.call[i],])

  }

  out<-list(assigned=ran.assign.call,prior = prior.assign, cause.true = tc)
  return(out)
}
```

The final two functions are used to estimate the cause-specific hazards using JAGS. The first function estimates the cause-specific hazards without including the observer's prior information for the sources of mortality for each animal, and the second includes that information in the estimation process.

```
certain.est<-function(cause.in,reps.in,datain1){
  datain1$cause<-cause.in
  jagsfit<- jags.parallel(datain1, init1, parameters.to.save=parms,
                          n.chains=3, n.iter=reps.in, model.file=model1,
                          n.burnin=0.1*reps.in, n.thin=1, DIC=FALSE,
                          working.directory=NULL)  #fit the model in JAGS
  j1<-as.mcmc(jagsfit)
  return(summary(j1))

}
uncertain.est<-function(prior.in,reps.in,datain2){
  datain2$p.obs<-prior.in
  jagsfit<- jags.parallel(datain2, init1, parameters.to.save=parms,
                          n.chains=3, n.iter=reps.in, model.file=model2,
                          n.burnin=0.1*reps.in, n.thin=1, DIC=FALSE,
                          working.directory=NULL)  #fit the model in JAGS
  j1<-as.mcmc(jagsfit)
  return(summary(j1))
}
```

The next step is to create the model used to by JAGS in estimation. The first model code is associated with estimating the cause-specific hazards without including the observer's prior information. In both models we use a generalized Bayes-Laplace prior (i.e., parameters of Dirichlet distribution $\overrightarrow{\alpha} = 1$) for the true probabilities of mortality arising from each source, and diffuse Normal priors for the log of the mortality hazard. The models are constructed to allow for staggered entry of individuals and interval censoring.

```
model1 <- function(){

  ###Model - Cause of death assigned with certainty

```

```
  # Priors
  llambda  ~ dnorm(0, 1.0E-6) # log hazard rate

  for(i in 1:4){
    alpha[i]<-1
  }

  p ~ ddirich(alpha[])  #true probability of cause of death

  # Likelihood for the total hazard
  for (j in 1:records) {
    for (k in left[j]:(right[j]-1)) {
      UCH[j,k] <- exp(llambda)
    }
    # total prob of surviving
    SLR[j] <- exp(-sum(UCH[j,left[j]:(right[j]-1)]))
    censor[j] ~ dbern(SLR[j])
  }



  # Likelihood cause of death
  for (i in 1:c.records) {
    cause[i] ~ dcat(p[])
  }

  #Derived parameters
  for (i in 1:4) {
    causehaz[i] <- exp(llambda)*p[i] #cause-specific hazards
  }
}
```

The second model is used to estimate the cause-specific hazards while including the observer's prior information. We employ the "zeros trick" to permit estimation.

```
model2<-function(){
  # Priors
  llambda  ~ dnorm(0, 1.0E-6) # log hazard rate

  for(i in 1:4){
    alpha[i]<-1
  }
  for(i in 1:c.records){
    cause[i] ~ dcat(p.obs[i,1:4])
  }

  #true probability of cause of death
  p ~ ddirich(alpha[])

  # Likelihood for the total hazard
  for (j in 1:records) {
    for (k in left[j]:(right[j]-1)) {
      UCH[j,k] <- exp(llambda)
    }
```

```
    # total prob of surviving
    SLR[j] <- exp(-sum(UCH[j,left[j]:(right[j]-1)]))
    censor[j] ~ dbern(SLR[j])
  }


  # Likelihood cause of death
  for (i in 1:c.records) {
    phi[i] <--log(p[cause[i]])

    #zeros trick since cause[i] given prior can't give ~ again
    z[i] ~ dpois(phi[i])
  }

  #Derived parameters
  for (i in 1:4) {
    causehaz[i] <- exp(llambda)*p[i] #cause-specific hazards
  }
}
```

We conclude the preliminary set up of the simulations by specifying the initial values and parameters we would like JAGS to monitor.

```
#specify initial values
init1<-function(){list("llambda"=-3, p=rep(0.25,4))}

#identify parms to monitor
parms<-c("llambda","p","causehaz")
```

The final section of code runs the actual simulations utilizing the functions created above. It begins with specifying the variable values that will be used in the simulations, and creating the array to hold the generated results.

```
sims.go<-function(p.in,name1,reps.ina){

  #sample size
  n<-50

  #number of months
  studylength<-36

  #constant hazard
  lambda<-0.1

  #multinomial probabilities for 4 death causes
  p<-p.in

  simreps<-reps.ina  #number of simulation reps
  results<-array(0,dim=c(simreps,36,31))  #array to hold results
  names1<-c("causehaz[1]", "causehaz[2]", "causehaz[3]",
            "causehaz[4]","llambda","p[1]","p[2]","p[3]","p[4]")
  outmu<-vector("character",length=length(names1))
  outsd<-vector("character",length=length(names1))
```

```
outlcl<-vector("character",length=length(names1))
outucl<-vector("character",length=length(names1))
for(i in 1:length(names1)){
  outmu[i]<-paste(names1[i],"mu",sep="")
  outsd[i]<-paste(names1[i],"sd",sep="")
  outlcl[i]<-paste(names1[i],"lcl",sep="")
  outucl[i]<-paste(names1[i],"ucl",sep="")
}

colnames(results)<-c(outmu,outsd,outlcl,outucl)
```

The next step is to begin the simulations and use a "for loop" to achieve the desired number of iterations. Within each iteration we create a unique dataset based on the parameters that we specified. Specifically, we generate a failure time from the exponential distribution with parameter, "lambda". We then create the observed data of entry time, last known alive, and first known dead times. This construct allows for staggered entry as well as interval censoring. Lastly, we organize the data for estimation in JAGS.

```
for(ii in 1:simreps){

  #####################Create Data

  #generate random failure times/add 1 since JAGS-no zero indices
  y<-rexp(n,lambda)+1

  #day of entry
  e<-rep(1,n)

  #last known alive
  r<-round(y,0)

  #first known dead
  s<-r+1


  ###Create data for hazard estimation in BUGS

  left<-c(e[r<=studylength],r[r<=studylength])
  right<-c(r[r<=studylength],s[r<=studylength])
  alive<-c(rep(1,sum(r<=studylength)),rep(0,sum(r<=studylength)))
  left<-c(left,e[r>studylength])  ##Add in rt. censored individuals
  right<-c(right,r[r>studylength])
  alive<-c(alive,rep(1,sum(r>studylength)))

  #remove survival pieces where die in first interval
  data<-cbind(left[left!=right],right[left!=right],
              alive[left!=right])
  records<-nrow(data)
  colnames(data)<-c("left","right","censor")
```

Next, we create the observer assigned causes of death, true cause of death and observer priors for the various mortality sources for the various confusion matrices .

```
#create assigned priors - classification 10
temp.result<-rassign(classm10,data[,"censor"],p)
prior1.data<-temp.result$prior
cause1<-temp.result$assigned
true.cause1<-temp.result$cause.true

#create assigned priors - classification 11
temp.result<-rassign(classm11,data[,"censor"],p)
prior2.data<-temp.result$prior
cause2<-temp.result$assigned
true.cause2<-temp.result$cause.true

#create assigned priors - classification 1
temp.result<-rassign(classm1,data[,"censor"],p)
prior3.data<-temp.result$prior
cause3<-temp.result$assigned
true.cause3<-temp.result$cause.true

#create assigned priors - classification 2
temp.result<-rassign(classm2,data[,"censor"],p)
prior4.data<-temp.result$prior
cause4<-temp.result$assigned
true.cause4<-temp.result$cause.true

#create assigned priors - classification 3
temp.result<-rassign(classm3,data[,"censor"],p)
prior5.data<-temp.result$prior
cause5<-temp.result$assigned
true.cause5<-temp.result$cause.true

#create assigned priors - classification 4
temp.result<-rassign(classm4,data[,"censor"],p)
prior6.data<-temp.result$prior
cause6<-temp.result$assigned
true.cause6<-temp.result$cause.true

#create assigned priors - classification 5
temp.result<-rassign(classm5,data[,"censor"],p)
prior7.data<-temp.result$prior
cause7<-temp.result$assigned
true.cause7<-temp.result$cause.true

#create assigned priors - classification 6
temp.result<-rassign(classm6,data[,"censor"],p)
prior8.data<-temp.result$prior
cause8<-temp.result$assigned
true.cause8<-temp.result$cause.true

#create assigned priors - classification 7
temp.result<-rassign(classm7,data[,"censor"],p)
prior9.data<-temp.result$prior
cause9<-temp.result$assigned
true.cause9<-temp.result$cause.true
```

```
#create assigned priors - classification 8
temp.result<-rassign(classm8,data[,"censor"],p)
prior10.data<-temp.result$prior
cause10<-temp.result$assigned
true.cause10<-temp.result$cause.true

#allow assignment based on prior.data
cause.na<-rep(NA,sum(data[,"censor"]==0))

#for zeros trick in JAGS
z<-rep(0,length(cause.na))
```

The final step before estimation is to format the data for input into JAGS.

```
###Estimation

datain1<-list(records=records, left=data[,"left"],
              right=data[,"right"], censor=data[,"censor"],
              cause=cause1,c.records=length(cause.na))

datain2<-list(records=records, left=data[,"left"],
              right=data[,"right"], censor=data[,"censor"],
              cause=cause.na,c.records=length(cause.na),z=z)
```

We conclude by estimating the parameters of interest using JAGS for the various confusion matrices for both models.

```
#biologists assignments -no priors
jagsfit1<-certain.est(cause1,15000,datain1)
results[ii,,1]<-c(as.vector(jagsfit1[[1]][,c("Mean","SD")]),
                  as.vector(jagsfit1[[2]][,c("2.5%","97.5%")]))



jagsfit2<-certain.est(cause2,15000,datain1)
results[ii,,2]<-c(as.vector(jagsfit2[[1]][,c("Mean","SD")]),
                  as.vector(jagsfit2[[2]][,c("2.5%","97.5%")]))

jagsfit3<-certain.est(cause3,15000,datain1)
results[ii,,3]<-c(as.vector(jagsfit3[[1]][,c("Mean","SD")]),
                  as.vector(jagsfit3[[2]][,c("2.5%","97.5%")]))

jagsfit4<-certain.est(cause4,15000,datain1)
results[ii,,4]<-c(as.vector(jagsfit4[[1]][,c("Mean","SD")]),
                  as.vector(jagsfit4[[2]][,c("2.5%","97.5%")]))

jagsfit5<-certain.est(cause5,15000,datain1)
results[ii,,5]<-c(as.vector(jagsfit5[[1]][,c("Mean","SD")]),
                  as.vector(jagsfit5[[2]][,c("2.5%","97.5%")]))

jagsfit6<-certain.est(cause6,15000,datain1)
results[ii,,6]<-c(as.vector(jagsfit6[[1]][,c("Mean","SD")]),
                  as.vector(jagsfit6[[2]][,c("2.5%","97.5%")]))
```

```
jagsfit7<-certain.est(cause7,15000,datain1)
results[ii,,7]<-c(as.vector(jagsfit7[[1]][,c("Mean","SD")]),
                  as.vector(jagsfit7[[2]][,c("2.5%","97.5%")]))

jagsfit8<-certain.est(cause8,15000,datain1)
results[ii,,8]<-c(as.vector(jagsfit8[[1]][,c("Mean","SD")]),
                  as.vector(jagsfit8[[2]][,c("2.5%","97.5%")]))

jagsfit9<-certain.est(cause9,15000,datain1)
results[ii,,9]<-c(as.vector(jagsfit9[[1]][,c("Mean","SD")]),
                  as.vector(jagsfit9[[2]][,c("2.5%","97.5%")]))

jagsfit10<-certain.est(cause10,15000,datain1)
results[ii,,10]<-c(as.vector(jagsfit10[[1]][,c("Mean","SD")]),
                   as.vector(jagsfit10[[2]][,c("2.5%","97.5%")]))


#biologists priors
jagsfit1a<-uncertain.est(prior1.data,15000,datain2)
results[ii,,12]<-c(as.vector(jagsfit1a[[1]][,c("Mean","SD")]),
                   as.vector(jagsfit1a[[2]][,c("2.5%","97.5%")]))

jagsfit2a<-uncertain.est(prior2.data,15000,datain2)
results[ii,,13]<-c(as.vector(jagsfit2a[[1]][,c("Mean","SD")]),
                   as.vector(jagsfit2a[[2]][,c("2.5%","97.5%")]))

jagsfit3a<-uncertain.est(prior3.data,15000,datain2)
results[ii,,14]<-c(as.vector(jagsfit3a[[1]][,c("Mean","SD")]),
                   as.vector(jagsfit3a[[2]][,c("2.5%","97.5%")]))

jagsfit4a<-uncertain.est(prior4.data,15000,datain2)
results[ii,,15]<-c(as.vector(jagsfit4a[[1]][,c("Mean","SD")]),
                   as.vector(jagsfit4a[[2]][,c("2.5%","97.5%")]))

jagsfit5a<-uncertain.est(prior5.data,15000,datain2)
results[ii,,16]<-c(as.vector(jagsfit5a[[1]][,c("Mean","SD")]),
                   as.vector(jagsfit5a[[2]][,c("2.5%","97.5%")]))

jagsfit6a<-uncertain.est(prior6.data,15000,datain2)
results[ii,,17]<-c(as.vector(jagsfit6a[[1]][,c("Mean","SD")]),
                   as.vector(jagsfit6a[[2]][,c("2.5%","97.5%")]))

jagsfit7a<-uncertain.est(prior7.data,15000,datain2)
results[ii,,18]<-c(as.vector(jagsfit7a[[1]][,c("Mean","SD")]),
                   as.vector(jagsfit7a[[2]][,c("2.5%","97.5%")]))

jagsfit8a<-uncertain.est(prior8.data,15000,datain2)
results[ii,,19]<-c(as.vector(jagsfit8a[[1]][,c("Mean","SD")]),
                   as.vector(jagsfit8a[[2]][,c("2.5%","97.5%")]))

jagsfit9a<-uncertain.est(prior9.data,15000,datain2)
results[ii,,20]<-c(as.vector(jagsfit9a[[1]][,c("Mean","SD")]),
                   as.vector(jagsfit9a[[2]][,c("2.5%","97.5%")]))
```

```
    jagsfit10a<-uncertain.est(prior10.data,15000,datain2)
    results[ii,,21]<-c(as.vector(jagsfit10a[[1]][,c("Mean","SD")]),
                       as.vector(jagsfit10a[[2]][,c("2.5%","97.5%")]))


    #no error in assignment
    jagsfit1b<-certain.est(true.cause1,15000,datain2)
    results[ii,,22]<-c(as.vector(jagsfit1b[[1]][,c("Mean","SD")]),
                       as.vector(jagsfit1b[[2]][,c("2.5%","97.5%")]))

    jagsfit2b<-certain.est(true.cause2,15000,datain2)
    results[ii,,23]<-c(as.vector(jagsfit2b[[1]][,c("Mean","SD")]),
                       as.vector(jagsfit2b[[2]][,c("2.5%","97.5%")]))

    jagsfit3b<-certain.est(true.cause3,15000,datain2)
    results[ii,,24]<-c(as.vector(jagsfit3b[[1]][,c("Mean","SD")]),
                       as.vector(jagsfit3b[[2]][,c("2.5%","97.5%")]))

    jagsfit4b<-certain.est(true.cause4,15000,datain2)
    results[ii,,25]<-c(as.vector(jagsfit4b[[1]][,c("Mean","SD")]),
                       as.vector(jagsfit4b[[2]][,c("2.5%","97.5%")]))

    jagsfit5b<-certain.est(true.cause5,15000,datain2)
    results[ii,,26]<-c(as.vector(jagsfit5b[[1]][,c("Mean","SD")]),
                       as.vector(jagsfit5b[[2]][,c("2.5%","97.5%")]))

    jagsfit6b<-certain.est(true.cause6,15000,datain2)
    results[ii,,27]<-c(as.vector(jagsfit6b[[1]][,c("Mean","SD")]),
                       as.vector(jagsfit6b[[2]][,c("2.5%","97.5%")]))

    jagsfit7b<-certain.est(true.cause7,15000,datain2)
    results[ii,,28]<-c(as.vector(jagsfit7b[[1]][,c("Mean","SD")]),
                       as.vector(jagsfit7b[[2]][,c("2.5%","97.5%")]))

    jagsfit8b<-certain.est(true.cause8,15000,datain2)
    results[ii,,29]<-c(as.vector(jagsfit8b[[1]][,c("Mean","SD")]),
                       as.vector(jagsfit8b[[2]][,c("2.5%","97.5%")]))

    jagsfit9b<-certain.est(true.cause9,15000,datain2)
    results[ii,,30]<-c(as.vector(jagsfit9b[[1]][,c("Mean","SD")]),
                       as.vector(jagsfit9b[[2]][,c("2.5%","97.5%")]))

    jagsfit10b<-certain.est(true.cause10,15000,datain2)
    results[ii,,31]<-c(as.vector(jagsfit10b[[1]][,c("Mean","SD")]),
                       as.vector(jagsfit10b[[2]][,c("2.5%","97.5%")]))

    print(ii)
  }
```

Results are saved for comparative analyses.

```
  saveRDS(results,name1)
}
```

We run this simulation for various values of the true probabilities of the cause of death.
