# Supplementary material for: Using expert knowledge to incorporate uncertainty in cause‐of‐death assignments for modeling of cause‐specific mortality
Source: Ecol Evol. 2017 Nov 30;8(1):509–20. doi: 10.1002/ece3.3701 (PMC5756890; doi:10.1002/ece3.3701)
Supplement: Supplementary file 2 [file ECE3-8-509-s002.docx]

**APPENDIX S1**

**Field Necropsy Fate Key – Assigning Prior Predictive Values (PP)**

Unnecessary if very confident or certain of cause, assign 1.0 to PP

Example legal harvest, dead on side of road, known predator kill of healthy deer

If additional lab necropsy is conducted use diagnosis to adjust PP

Only select a single value from any cause-specific fate

Do not select any value if cause is not suspected

When finished with field necropsy and completed all datasheets:

If there is only one cause suspected assign PP = 1.0

If multiple causes are suspected divide each cause-specific score by total summed scores to assign PP

**Wolf Predation**

**cause-specific score / ∑all causes( ) = _____**

Confident in wolf predation 90

Small chance of another contributing cause/stress

Likely wolf predation involved 70

Decent chance of another contributing cause/stress

Some chance wolf predation involved 30

Good chance of another contributing cause/stress

Slight chance wolf predation involved 10

Very likely chance of another contributing cause/stress

**Coyote Predation**

**cause-specific score / ∑all causes( ) = _____**

Confident in coyote predation 90

Small chance of another contributing cause/stress

Likely coyote predation involved 70

Decent chance of another contributing cause/stress

Some chance coyote predation involved 30

Good chance of another contributing cause/stress

Slight chance coyote predation involved 10

Very likely chance of another contributing cause/stress

**Bobcat Predation**

**cause-specific score / ∑all causes( ) = _____**

Confident in bobcat predation 90

Small chance of another contributing cause/stress or unknown

Likely bobcat predation involved 70

Decent chance of another contributing cause/stress or unknown

Some chance bobcat predation involved 30

Good chance of another contributing cause/stress or unknown

Slight chance bobcat predation involved 10

Very likely chance of another contributing cause/stress or unknown

**Other Predation**

**cause-specific score / ∑all causes( ) = _____**

Confident in other predation 90

Small chance of another contributing cause/stress

Likely other predation involved 70

Decent chance of another contributing cause/stress

Some chance other predation involved 30

Good chance of another contributing cause/stress

Slight chance other predation involved 10

Very likely chance of another contributing cause/stress

**Starvation (if back fat or organ fat is available use that as primary indication of nutritional status)**

**cause-specific score / ∑all causes( ) = _____**

Reddish/Clear Jelly throughout femur marrow (<5% fat) 70

No sign of any other cause/stress, but not convinced of fate

Other evidence of starvation at that time of year

Or other contributing factors to starvation (e.g., young-of-year)

Reddish/Clear Jelly throughout femur marrow (<5% fat) 50

Some sign of any other cause/stress

Other evidence of starvation at that time of year

Or other contributing factors to starvation (e.g., young-of-year)

Reddish/Clear Jelly through some femur marrow (5-15% fat) 50

No sign of any other cause/stress, but not convinced of fate

Other evidence of starvation at that time of year

Or other contributing factors to starvation (e.g., young-of-year)

Reddish/Clear Jelly through some femur marrow (5-15% fat) 30

Some sign of any other cause/stress

Other evidence of starvation at that time of year

Or other contributing factors to starvation (e.g., young-of-year)

No femur or any other long bone available 70

Notable starvation during same time in other radiocollared deer

No sign of additional causes/stress

No femur or any other long bone available 60

Very thin or some indication of starvation (documented starv same time, YOY, etc)

No sign of additional causes/stress

No femur or any other long bone available 40

Very thin or some indication of starvation (documented starv same time, YOY, etc)

Some sign of additional causes/stress

Femur or body condition indicate malnourished deer 5

During a time when starvation is non-existent in other deer (summer, very mild winter)

**Deer-vehicle-collision**

**cause-specific score / ∑all causes( ) = _____**

Within 200 yards of road 95

Crushed or broken bones likely related to DVC, but not convinced of fate

Within 200 yards of relatively busy road and vehicle speeds >55mph 90

Possibly crushed or broken bones related to DVC

Within 200 yards of relatively busy road and vehicle speeds 30-50mph 80

Possibly crushed or broken bones related to DVC

Within 200 yards of less busy paved road and vehicle speeds >55mph 80

Possibly crushed or broken bones related to DVC

Within 200 yards of less busy road and vehicle speeds 30-50mph 70

Possibly crushed or broken bones related to DVC

Within 50 yards of relatively busy road and vehicle speeds >55mph 90

No sign of crushed or broken bones

No sign of additional causes/stress

Within 50 yards of relatively busy road and vehicle speeds 30-50mph 80

No sign of crushed or broken bones

No sign of additional causes/stress

Within 50 yards of less busy paved road and vehicle speeds >55mph 85

No sign of crushed or broken bones

No sign of additional causes/stress

Within 50 yards of less busy road and vehicle speeds 30-50mph 75

No sign of crushed or broken bones

No sign of additional causes/stress

Within 100 yards of relatively busy road and vehicle speeds >55mph 80

No sign of crushed or broken bones

No sign of additional causes/stress

Within 100 yards of relatively busy road and vehicle speeds 30-50mph 70

No sign of crushed or broken bones

No sign of additional causes/stress

Within 100 yards of less busy paved road and vehicle speeds >55mph 75

No sign of crushed or broken bones

No sign of additional causes/stress

Within 100 yards of less busy road and vehicle speeds 30-50mph 65

No sign of crushed or broken bones

No sign of additional causes/stress

Within 200 yards of relatively busy road and vehicle speeds >55mph 60

No sign of crushed or broken bones

No sign of additional causes/stress

Within 200 yards of relatively busy road and vehicle speeds 30-50mph 50

No sign of crushed or broken bones

No sign of additional causes/stress

Within 200 yards of less busy paved road and vehicle speeds >55mph 55

No sign of crushed or broken bones

No sign of additional causes/stress

Within 200 yards of less busy road and vehicle speeds 30-50mph 45

No sign of crushed or broken bones

No sign of additional causes/stress

**Legal Reported Harvest**

**cause-specific score / ∑all causes( ) = _____**

Collar found with or near deer and deer hunter 95

Evidence that deer was going to be registered within time limit

Slight suspicion of non-compliance

Collar found with or near deer and deer hunter (see poaching section) 80

Evidence that deer was going to be registered within the time limit

Slight evidence of illegal activity and/or non-compliance

Collar found with or near deer and deer hunter (see poaching section) 70

Slight evidence that deer was going to be registered

Some evidence of illegal activity and/or non-compliance

Collar found with or near deer and deer hunter (see poaching section) 30

Suspicion that deer was not going to be registered

Or notorious for non-compliance or poaching

Missing or cut collar during hunting season 30

Add 5 if primarily on private land (assuming low poach rate)

Subtract 10-20 if some suspicion of poaching (attempts to hide collar, noncompliance)

Subtract 10-20 if MIA and battery may have died or dispersal time

**Wounding Loss**

**cause-specific score / ∑all causes( ) = _____**

Bullet or arrow wound during legal season 95

Likely mortal (Infected or excessive bleeding)

No human sign, but not convinced of fate

Bullet or arrow wound with some bleeding during legal season 90

Could be mortal

No human sign

Bullet or arrow wound with excessive bleeding during legal season (see poaching) 60

Likely mortal

Slight chance or evidence hunter saw dead deer and decided not to tag

Unable to determine if wounded but heavy hunting pressure during legal season 20

**Poaching**

**cause-specific score / ∑all causes( ) = _____**

Bullet or arrow wound with excessive bleeding outside of legal season 95

Likely mortal wound, but not convinced of fate

Bullet or arrow wound healing over and/or min. infection outside legal season 80

Not likely mortal wound, but infection or stress caused mortality

High prevalence of poaching in area outside of legal season 10

Unable to determine if wounded

Bullet or arrow wound with excessive bleeding during legal season 80

Very good chance or evidence hunter saw dead deer and decided not to tag

Bullet or arrow wound with excessive bleeding during legal season 70

Good chance or evidence hunter saw dead deer and decided not to tag

Bullet or arrow wound with excessive bleeding during legal season 60

Some chance or evidence hunter saw dead deer and decided not to tag

Missing or cut collar during hunting season 50

Subtract 5 if primarily on private land (assuming lower poach rate)

Add 10-30 if some suspicion of poaching (attempts to hide collar, noncompliance)

Subtract 10-30 if MIA and battery may have died or dispersal time

Potential cut collar found outside of hunting season 60

Slight evidence of human killed deer

Little potential for other cause or stress

Potential cut collar found outside of hunting season 30

Minimal evidence of human killed deer

Some potential for other cause or stress

**Other – (e.g., Disease, Other Trauma)**

**cause-specific score / ∑all causes( ) = _____**

Evidence that the deer was compromised more than most deer 90

e.g. infection, injury, worn down/missing teeth, lots of parasites

Little chance other contributing factors from above causes or not additional evidence

Evidence that the deer was compromised more than most deer 50

e.g. infection, injury, worn down/missing teeth, lots of parasites

Some chance other contributing factors from above causes or not additional evidence

Evidence that the deer was compromised more than most deer 20

e.g. infection, injury, worn down/missing teeth, lots of parasites

Decent chance other contributing factors from above causes or not additional evidence
